# Development of a social vulnerability index: Enhancing approaches to support climate justice

**DOI:** 10.1016/j.mex.2025.103290

**Published:** 2025-03-26

**Authors:** D McCullagh, W Cámaro-García, D Dunne, P Nowbakht, L Cumiskey, C Gannon, C Phillips

**Affiliations:** a MaREI, the SFI Research Centre for Energy, Climate and Marine; b Department of Geography, University College Cork, Cork, Ireland; c Environmental Research Institute, University College Cork, Cork, Ireland

**Keywords:** Social vulnerability, Climate change, Environmental hazards, Just transition, Climate adaptation decision-making, Social Vulnerability Index

## Abstract

Climate change is causing increasing frequency and severity of various hazards such as flooding and extreme temperatures. Vulnerability analysis that broadens the focus beyond exposure to hazards is invaluable in supporting just climate action. This study outlines the modifications made to the social vulnerability to environmental hazards index developed by Fitton et al., [1] building upon previous work to make the index hazard specific and applicable across a range of locations, with case studies in Ireland, Italy, Northern Ireland and Spain and by a variety of users. New indicators have been included in the current version of the Social Vulnerability Index (SVI) and various weighting methods proposed. This method was developed using programming tools in R and GIS (Geographic Information Systems), both of which are accessible and easily adapted and updated, to support wider dissemination and overall usability.

• Step-by-step guidance on the use of the SVI so that the method can be adapted and replicated
• A variety of methodology options to suit users with different levels of data availability
• Tailored to the needs of local authorities to support climate adaptation measures that are equitable

## Graphical-abstract

**Figure:**
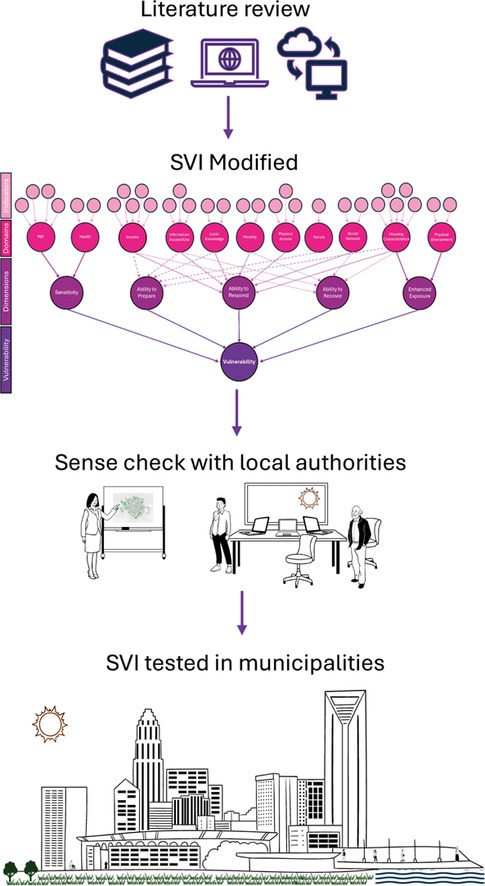

Specifications table

**Table:** 

Subject area:	Environmental Science
More specific subject area:	*Climate Change Adaptation – Informing equitable decision-making*
Name of your method:	*Social Vulnerability Index*
Name and reference of original method:	*Fitton, J.M., O'Dwyer, B. & Maher, B. (2021) Developing a social vulnerability to environmental hazards index to inform climate action in Ireland. Irish Geography, Vol. 54, 2. DOI:*10.2014/igj.v54i2.1468
Resource availability:	*Zenodo Databases for Cork [*https://doi.org/10.5281/zenodo.13913850]*, Logroño, [*https://doi.org/10.5281/zenodo.13909225*] & Milan [*https://doi.org/10.5281/zenodo.13894260*]; Jupyter Book [*https://ucc-climpadap.github.io/SVIBook/*], GitHub Repository [*https://github.com/UCC-CLIMPADAP/SVIBook*]*

## Background

Climate change is leading to increasingly extreme weather events, from flooding to heatwaves and storms [2]. These weather events are hazardous to communities and organisations, causing loss of life, disrupting day-to-day operations, causing damage to assets and infrastructure and subsequent economic losses across cities and regions [3,4]. The vulnerability of people and communities to such events can often depend on factors beyond the physical hazard itself. Hurricane Katrina illustrated how the same climatic event is experienced in different ways by different people, with outcomes varying across geographic and socioeconomic gradients [5]. By considering the social dimension of an area when mapping vulnerability, decision-makers can ensure that subsequent climate adaptation action does not exacerbate existing inequalities [6]. As outlined by Fitton et al. [1], who designed the first iteration of the social vulnerability to environmental hazards index, vulnerability is considered as “*the sensitivity of a population to natural hazards and its ability to respond to and recover from the impacts of hazards*”. The aim of many councils and local authorities is to challenge social disadvantage and marginalisation of people and communities within their area. The Social Vulnerability Index (SVI) has the potential to apply the theoretical ideas of social vulnerability into an approach that can provide baseline information on the different social dimensions at play within different localities and regions, beyond the Irish context. This can inform the design of locally relevant and socially just climate adaptation policy and action, helping authorities meet local and national goals to achieve a just transition.

Using an indicator approach and high-resolution information can provide an accurate estimation of baseline vulnerability at the local level, which is essential for decision makers to reduce climate impacts and disaster risk [7,8]. When addressing climate hazards, a ‘*one size fits all’* approach will not work, as the impacts of hazards are often place specific. By working in partnership with local municipalities, recent developments in the SVI allow for the nuances within local contexts to be considered and included, ensuring more robust outputs. Following on from the work by Fitton et al. [1], the key objectives of this research using the now renamed Social Vulnerability Index (SVI) include:

• mapping the vulnerability of different areas across Europe to the specific climate hazards of extreme heat and flooding;
• updating the previous methodology to allow the tool to be applied in different spatial, environmental and decision-making contexts;
• providing detailed user guidance to ensure that the tool can applied by local authorities and integrated with additional climate services to provide real-world applications and more detailed outputs for users to address climate challenges in their areas.

## Method details

This work has been carried out primarily through the REACHOUT project, which aims to advance user orientated climate services and support the implementation of the European Green Deal. A key element of this work was the tailoring of climate services and tools to the needs of the seven city hubs involved in the project. After discussions with city stakeholders, representatives from Cork City, Ireland, Logroño, Spain and Milan, Italy all expressed an interest in co-developing the SVI tool to support wider climate justice priorities in their cities. Following the interest of the SVI tool in these cities, the opportunity to utilise it was also extended more widely to other local and regional authorities through the Directed and TALX2 projects, where the authors are currently engaged in climate impacts and adaptation research with local authorities and communities. The Directed project aims to reduce vulnerability to extreme weather events and foster disaster-resilient European societies, with four real-world labs located across Europe, including one in Rimini, Italy, whose city representatives wished to better understand the climate risk to specific vulnerable populations. The TALX2 project aims to support and progress just climate adaptation activities on the island of Ireland using place-based partnerships and has established living labs and a cross-border programme of activities to support this. As a part of this work, representatives of Derry City and Strabane District Council and Belfast City Council have expressed an interest in developing the SVI for the entire region of Northern Ireland to support equitable adaptation action here. This study builds on the method proposed by Fitton et al. [1], which was based upon the methodologies of Kazmierczak et al. [9], Lindley & O'Neil [10], and Lindley et al. [11] and focused on social vulnerability to environmental hazards in Ireland. The different domains and their relationship with social vulnerability to climate hazards outlined in both the Fitton et al. [1] methodology and this research, are well established in international literature [5,9,[12], [13], [14], [15], [16], [17], [18]]. The updated methodology focuses on the specific hazards of flooding and extreme heat that are key issues for the aforementioned areas, rather than the more general environmental hazards used by Fitton et al [1] and proposes a set of potential indicators derived through the exploration of the most up to date national census data in each of the regions and European Copernicus data. Additionally, through the experience of working with city representatives in Cork, Logroño and Milan the original methodology was found to be insufficient in some areas due to the data availability in some of the cities.

**Fig. 1:**
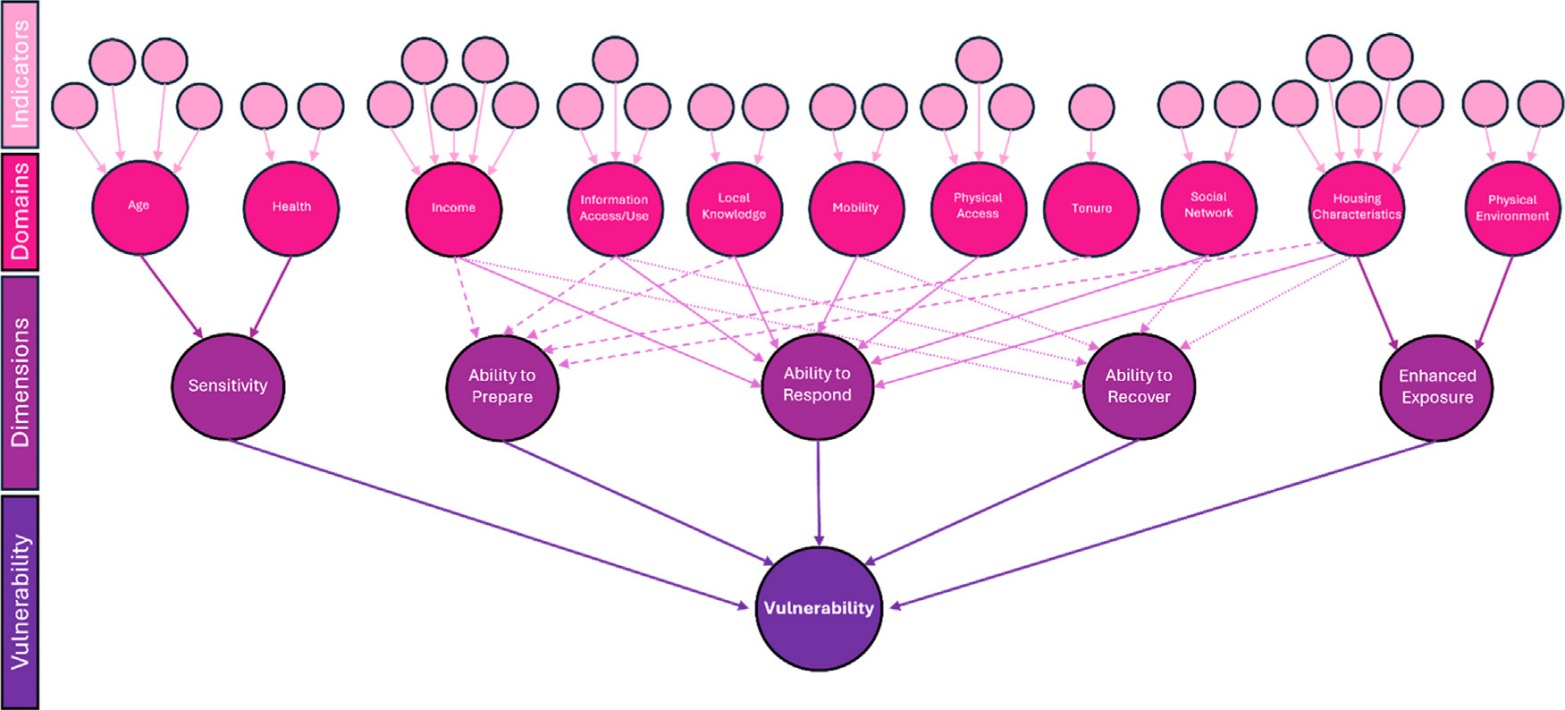
Graphic representation of the methodology used to create the domains, dimensions, and overall vulnerability in Cork City (adapted from Fitton et al., 2021).

As proposed in the original method [1] indicators are integrated into a hierarchy scheme. Indicators are grouped into domains, with Z-scores for each domain summed with an equal weighting for the spatial area used. The domains are then collated and associated with dimensions of social vulnerability (sensitivity, adaptive capacity and enhanced exposure). Through this research, changes were made to the methodology to ensure it was better tailored to the specific hazards of either flooding or extreme heat, relevant in each area, based on the data available. New indicators were introduced, such as those for volunteering and unoccupied dwellings, while indicators in some domains, such as housing characteristics, were found to contribute to additional dimensions. For example, specific housing characteristic indicators such as households with no central heating and households with private water supplies related better to adaptive capacity in terms of flooding than to enhanced exposure, and where therefore moved to reflect this (Fig. 1).

This update to the original methodology, with the inclusion of additional flood specific indicators (Table 1) applied well in Cork City, building a robust SVI score. However, when the authors attempted to apply this same methodology outside of Ireland, in Logroño and Milan, they encountered problems due to the availability of data. Data collection and publication for the national census can differ significantly by country, with different data collection methods, varying time periods for processing, and aggregation of the data at different spatial resolution, dependent upon national resource and General Data Protection Regulation (GDPR) policies. In the case of both Logroño and Milan, some of the domains outlined in Fig. 1, did not have available indicator data, with the tables below highlighting the different levels of census data availability in different national contexts and the rationale behind specific indicator selection (Table 1, Table 2–3).

**Table 1:** Indicators available in Cork City, Ireland to determine vulnerability to flooding (* indicators are additional to those used by Fitton et al [1]) (taken from the national census for Ireland and from European Copernicus datasets on land cover).

Indicator	Domain	Dimension	Rationale
Children under 5 years of age	Age	Sensitivity	Physiologically, the young have a greater susceptibility to the effects of flooding.
Adults over 75 years of age	Age	Sensitivity	Physiologically, older people have a greater susceptibility to the effects of flooding.
Persons with poor health	Health	Sensitivity	Physiologically, those with an illness have a greater susceptibility to the effects of flooding.
People with a disability preventing work	Health	Sensitivity	Physiologically, those with a disability have a greater susceptibility to the effects of flooding.
One parent households	Income	Adaptive Capacity – Ability to Prepare/Respond/Recover	One parent households are more likely to have less disposable income compared to other households
*Children per family	Income	Adaptive Capacity – Ability to Prepare/Respond/Recover	People with more dependents may struggle to respond to and recover from extreme climatic events.
Low skilled employment	Income	Adaptive Capacity – Ability to Prepare/Respond/Recover	People in low skills employment are likely to have less income, reducing their ability to adapt before, during, or after an event.
Population employed in farming	Income	Adaptive Capacity – Ability to Prepare/Respond/Recover	People who are work with farming related occupations are potentially more impacted by hazards due to the reliance upon access and quality of farmland
Unemployment	Income	Adaptive Capacity – Ability to Prepare/Respond/Recover	Unemployed persons are more likely to have less incomes, and have limited ability to make physical adjustments to their property to adapt to flooding
*Population with no higher education	Information Access/Use	Adaptive Capacity – Ability to Prepare/Respond/Recover	People with limited education may find it difficult to interpret and/or act up on information received
Population who do not speak English well or at all	Information Access/Use	Adaptive Capacity – Ability to Prepare/Respond/Recover	People with poor English ability may find it difficult to interpret and/or act up on information received
Households with no Internet	Information Access/Use	Adaptive Capacity – Ability to Prepare/Respond/Recover	People with households with no internet may find it difficult to find information, pre, during, and post flooding events
New residents	Local Knowledge	Adaptive Capacity – Ability to Prepare/Respond	Residents living in the area less than a year are likely to have less local knowledge and may be less aware of hazards.
*Foreign nationals	Local Knowledge	Adaptive Capacity – Ability to Prepare/Respond	Foreign nationals are likely to have less local knowledge and may be less aware of hazards.
Households with no motor car	Mobility	Adaptive Capacity – Ability to Respond/Recover	No personal access to a vehicle may restrict evacuation during a flooding event
Travel Time	Physical Access	Adaptive Capacity – Ability to Respond	People with long commutes to work/ school are likely living in areas with low service provision
Households renting	Tenure	Adaptive Capacity – Ability to Prepare	Renters are more likely to have lower incomes, plus have limited ability to make physical adjustments to their property to adapt to flooding.
*Volunteering	Social Network	Adaptive Capacity – Ability to Respond/Recover	Those engaged in volunteering tend to have a stronger social network that can assist them during a flooding event.
Primary school age children	Social Network	Adaptive Capacity – Ability to Respond/Recover	Those with primary school age children tend to have a stronger social network that can assist them during a flooding event.
Households with one person	Social Network	Adaptive Capacity – Ability to Respond/Recover	Those living alone likely lack a support network that can assist them during a flooding event.
Households with no central heating	Housing Characteristics	Adaptive Capacity – Ability to Recover	People that reside in households with no central heating may struggle to recover from the impacts of flooding events (cold and damp)
Households with private water supplies	Housing Characteristics	Adaptive Capacity – Ability to Recover	A private water supply may not have as sophisticated water quality monitoring as compared to the public supply, making the occurrence of water borne diseases more likely after a flooding event
Dwelling construction year	Housing Characteristics	Enhanced Exposure	Households built prior to 1945 may be of poor quality and may not offer as much protection to residents when compared to modern housing
Households that are caravans/mobile homes	Housing Characteristics	Enhanced Exposure	Caravans/mobile homes may not offer as much protection to residents as compared to permanent housing
*Unoccupied dwellings	Housing Characteristics	Enhanced Exposure	Unoccupied dwellings indicate that residents are away and may prevent deployment of cautionary measures such as door guards or sandbags during a flooding event.
Impervious surface	Physical Environment	Enhanced Exposure	Increased area of impervious surface enhances the impacts of flooding
*Tree cover	Physical Environment	Enhanced Exposure	Increased area of tree cover and greenspace reduces the impacts of flooding

The original methodology [1] assigned equal weights to each domain and distributed the weight based on the number of indicators for each domain. These domains were collated in dimensions and combined to form the overall social vulnerability index. In this way, each of the domains was considered an important element for determining social vulnerability and each indicator has an equal value in the relevant domain. The lack of data in Logroño and Milan for some domains and dimensions meant that some domains were missing, or that a single indicator could potentially be representing an entire domain or dimension, creating an unequal and disproportionate distribution of weighting in the SVI score.

**Table 2:** Indicators available in Logroño, Spain, to determine vulnerability to extreme heat (taken from the national census for Spain and from European Copernicus datasets on land cover).

Indicator	Domain	Dimension	Rationale
Boys under 5 years of age	Age	Sensitivity	Physiologically, the young have a greater susceptibility to the effects of extreme heat.
Girls under 5 years of age	Age	Sensitivity	Physiologically, the young have a greater susceptibility to the effects of extreme heat.
Males over 75 years of age	Age	Sensitivity	Physiologically, older people have a greater susceptibility to the effects of extreme heat.
Females over 75 years of age	Age	Sensitivity	Physiologically, older people have a greater susceptibility to the effects of extreme heat.
People with a disability preventing work	Health	Sensitivity	Physiologically, those with a disability have a greater susceptibility to the effects of extreme heat.
One parent households	Income	Adaptive Capacity – Ability to Prepare/Respond/Recover	One parent households are more likely to have less disposable income compared to other households
Dependents rate	Income	Adaptive Capacity – Ability to Prepare/Respond/Recover	People with dependents may struggle to respond to and recover from extreme climatic events
Unemployment	Income	Adaptive Capacity – Ability to Prepare/Respond/Recover	Unemployed persons are more likely to have less incomes, and have limited ability to make physical adjustments to their property to adapt to extreme heat
Population attending university	Income	Adaptive Capacity – Ability to Prepare/Respond/Recover	Students are more likely to have lower incomes, plus have limited ability to make physical adjustments to their property to adapt to extreme heat
Population with no higher education	Information Access/Use	Adaptive Capacity – Ability to Prepare/Respond/Recover	People with no formal education may find it difficult to interpret and/or act up on information received
Percentage of foreign nationals	Local Knowledge	Adaptive Capacity – Ability to Prepare/Respond	Foreign nationals are likely to have less local knowledge and be less aware of hazards
Households renting	Tenure	Adaptive Capacity – Ability to Prepare	Renters are more likely to have lower incomes, plus have limited ability to make physical adjustments to their property to adapt to extreme heat
Households with one person	Social Network	Adaptive Capacity – Ability to Respond/Recover	Those living alone likely lack a support network that can assist them during a heatwave event
Primary school age children	Social Network	Adaptive Capacity – Ability to Respond/Recover	Those with primary school age children tend to have a stronger social network that can assist them during a heatwave event
Dwelling construction year	Housing Characteristics	Enhanced Exposure	Households built prior to 1970 may be of poor quality and may not offer as much protection to residents as compared to modern housing
Impervious surface	Physical Environment	Enhanced Exposure	Increased area of impervious surface enhances the urban heat island affect
Tree cover	Physical Environment	Enhanced Exposure	Increased area of tree cover and greenspace reduces the urban heat island affect

**Table 3:** Indicators available in Milan, Italy, to determine vulnerability to extreme heat (taken from the national census for Italy and from European Copernicus datasets on land cover).

Indicator	Domain	Dimension	Rationale
Boys under 5 years of age	Age	Sensitivity	Physiologically, the young have a greater susceptibility to the effects of extreme heat.
Girls under 5 years of age	Age	Sensitivity	Physiologically, the young have a greater susceptibility to the effects of extreme heat.
Males over 75 years of age	Age	Sensitivity	Physiologically, older people have a greater susceptibility to the effects of extreme heat.
Females over 75 years of age	Age	Sensitivity	Physiologically, older people have a greater susceptibility to the effects of extreme heat.
Dependents rate	Income	Adaptive Capacity – Ability to Prepare/Respond/Recover	People with dependents may struggle to respond to and recover from extreme climatic events
Unemployment	Income	Adaptive Capacity – Ability to Prepare/Respond/Recover	Unemployed persons are more likely to have less incomes, and have limited ability to make physical adjustments to their property to adapt to extreme heat
Population with no higher education	Information Access/Use	Adaptive Capacity – Ability to Prepare/Respond/Recover	People with no formal education may find it difficult to interpret and/or act up on information received
Percentage of foreign nationals	Local Knowledge	Adaptive Capacity – Ability to Prepare/Respond	Foreign nationals are likely to have less local knowledge and be less aware of hazards
Primary school age children	Social Network	Adaptive Capacity – Ability to Respond/Recover	Those with primary school age children tend to have a stronger social network that can assist them during a heatwave event
Households with one person	Social Network	Adaptive Capacity – Ability to Respond/Recover	Those living alone likely lack a support network that can assist them during a heatwave event
Impervious surface	Physical Environment	Enhanced Exposure	Increased area of impervious surface enhances the urban heat island affect
Tree cover	Physical Environment	Enhanced Exposure	Increased area of tree cover and greenspace reduces the urban heat island affect

**Fig. 2:**
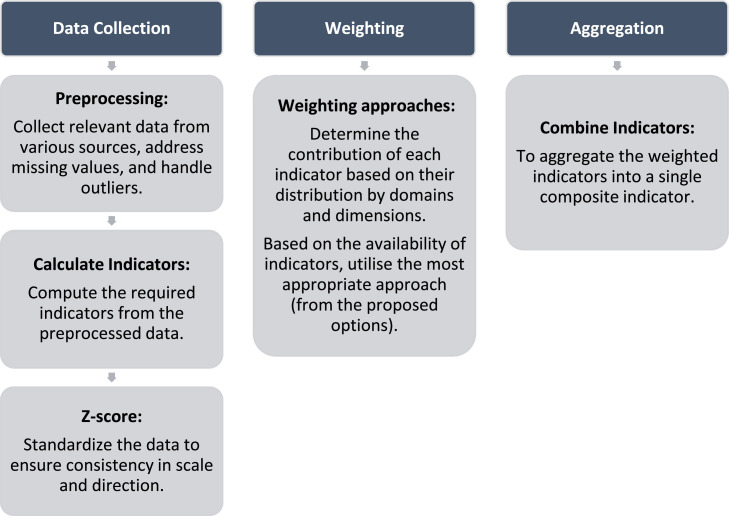
Process overview for calculating the Social Vulnerability Index (SVI) score.

To account for different data availability and accessibility, the authors adapted the original methodology using the process outlined in Fig. 2. This was to support the needs of all urban stakeholders and to ensure that the SVI methodology was as robust as possible for cases with differing levels of data availability. This also allows users in areas with lower data availability to easily identify the domains and dimensions where new indicators need to be added through local data collection or acquired in future census campaigns.

While a variety of potential weighting options (such as z-score, Ranking, Min–max, Distance from the group leader, Division by total, Categorical scale, etc.) can be used, the weighting option chosen for those areas with high levels of data available in all domains (i.e. Cork), was the z-score with equal weighting method for each domain, whilst for the cases with a lack of data, an easy to follow z-score with conditional approach based on the relationship between domains and dimensions was proposed. This option was discussed with the city representatives based on their need of interpretability of each domain and indicator as a single stand-alone product and considering the small number of indicators available for domains in some case studies. This methodology was considered by the authors and city representatives to be the least complicated to use and therefore its selection was to ensure ease of use by nontechnical audiences, aligning with the primary goal of the study to ensure the long-term usability of the SVI by local authorities, who may not have a high level of technical expertise.

In addition to the cases analysed under this study, the authors proposed an additional weighting methodology for cases where the information available is not representing all the dimensions under the concept of vulnerability. For these cases, the weighting option proposed is the Principal Component Analysis (PCA) applied over the indicators available, ignoring the aggregation of indicators to domains and dimensions. This methodology does not allow the indicators to be viewed as a single stand-alone product but allows the stakeholders to develop a robust index with the information available. This index is not assessing the vulnerability and new indicators are needed to cover the full vulnerability concept, but it could be used as a reference for decision making processes.

A detailed description of all the phases as showed in Fig. 2 is presented below. The data collection and pre-processing data is a key part of the process, translating the raw information collected from the data sources into standardized indicators comparable between them. Weighting and aggregation are fundamental steps in constructing composite indices, such as the SVI. Weighting assigns relative importance to each indicator, reflecting its contribution to the overall index. Aggregation combines these weighted indicators into a single composite score, summarising the multidimensional data into a comprehensible measure.

### Data collection

1. Begin gathering relevant data from multiple sources, including census data, health records, and environmental datasets, to address various social and economic factors that influence vulnerability, such as income, education, housing quality, and access to healthcare (see supplementary material for potential indicators). Once collected, the data should be cleaned by addressing missing values, removing or correcting outliers, and ensuring consistency. This may involve converting units, standardising variable formats, or geocoding records so that all information aligns with the same geographical units, such as small areas or census tracts
2. After preprocessing, specific indicators representing different aspects of social vulnerability should be computed; for example, the percentage of the population without higher education or the proportion of one-parent households.
3. In the process of calculating the SVI, z-score normalisation ensures that indicators measured on different scales become comparable. This step adjusts the values so that each indicator has the same magnitude and direction, meaning that higher values consistently reflect higher vulnerability (or vice versa). The z-score technique standardises data based on mean and standard deviation, resulting in a distribution with a mean of 0 and a standard deviation of 1, by applying the Eq. 1:
  (1) $$Z_{score}=\;\frac{x-\mu \;}{\sigma }$$

where, x represents the original value, µ is the mean of data and σ is the standard deviation of data.

### Weighting approaches

Based on the availability of data and indicators for the different case studies and in how these are grouped into several domains, a new set of weighted approaches, are. This allows users to avoid issues where missing data can cause bias in the final vulnerability score. For example, if in the age domain, key information such as the population under <5 is missing, the only indicator left, population >75, would control the domain, unbalancing the domain and potentially the dimension, and creating an overall z-score of vulnerability that will be biased towards a single indicator.

To avoid bias as much as possible, at least one indicator providing information on the dimensions of sensitivity, adaptive capacity (including ability to prepare, ability to respond, ability to recover) and enhanced exposure, was considered necessary for the creation of an SVI score. Wherever possible, providing a range of indicators for each domain (e.g. >75 years old male and female and <5 years old male and female for the age domain) was considered the best approach, allowing for a more robust overall vulnerability score and subsequent map output.

To avoid disproportionate weights, a set of tiered rules and methodologies are proposed by the authors. This ensures that all municipal actors and organisations can still avail themselves of the SVI tool, even where there is a lack of available data for all indicators. The rules are based on the different case studies explored in the REACHOUT project and can be used as guidance in deciding appropriate weighting methodologies for other locations.

#### Tiered weighting methodologies proposed

Based on the information available for the cities under analysis and the limitations related to the number of indicators and domains in some areas, we propose a set of tiered methodologies in the calculation of the final vulnerability index.

**Fig. 3:**
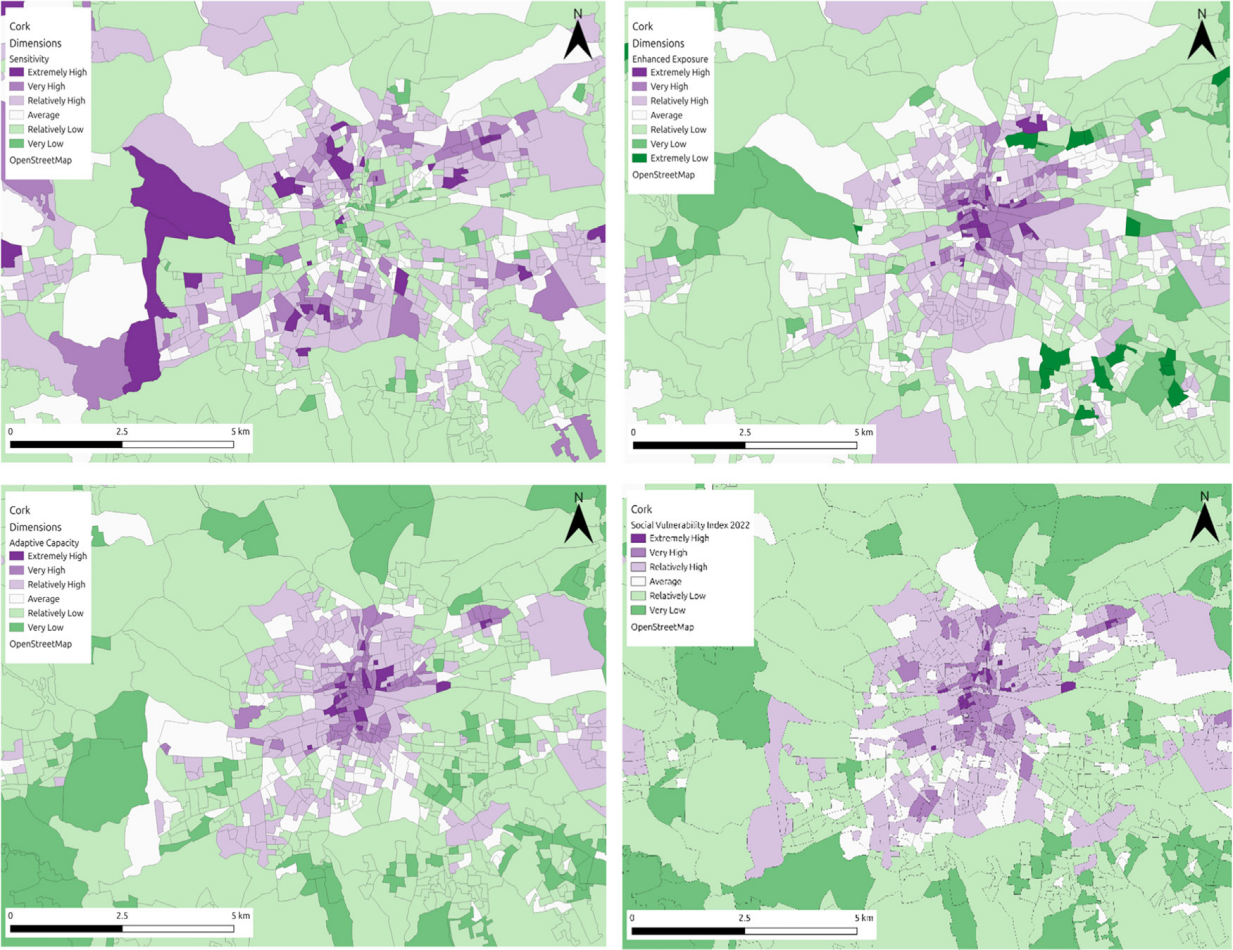
Visual representation of the SVI for flooding in Cork, alongside dimension specific representations (top left: sensitivity; bottom left: adaptive capacity; top right: enhanced exposure; bottom right: overall vulnerability).

*Tier 1*: This case represents the ideal scenario (Fig. 3) where there are at least two domains per dimension and each domain contains indicators representing the whole population vulnerable to the hazard. For this scenario we propose estimating the weights coefficients based on the number of indicators of each domain. Then for each domain the coefficients are equally distributed between the indicators and the sum of those are equal to 1.

The whole vulnerability index is then estimated aggregating all the relevant indicators with the weights coefficients estimated through the domains. See Eq. 2.

(2) $$SVI=\;\sum_{1}^{n}\sum_{1}^{m}W_{mn}*ind_{m}$$

$$W_{mn}=\;1/m$$

Where SVI is the vulnerability index, *ind_m_* is the m^th^ indicator, m is the total number of indicators for each n domain, and W_mn_ is the weight of the m^th^ indicator for the n^th^ domain.

**Fig. 4:**
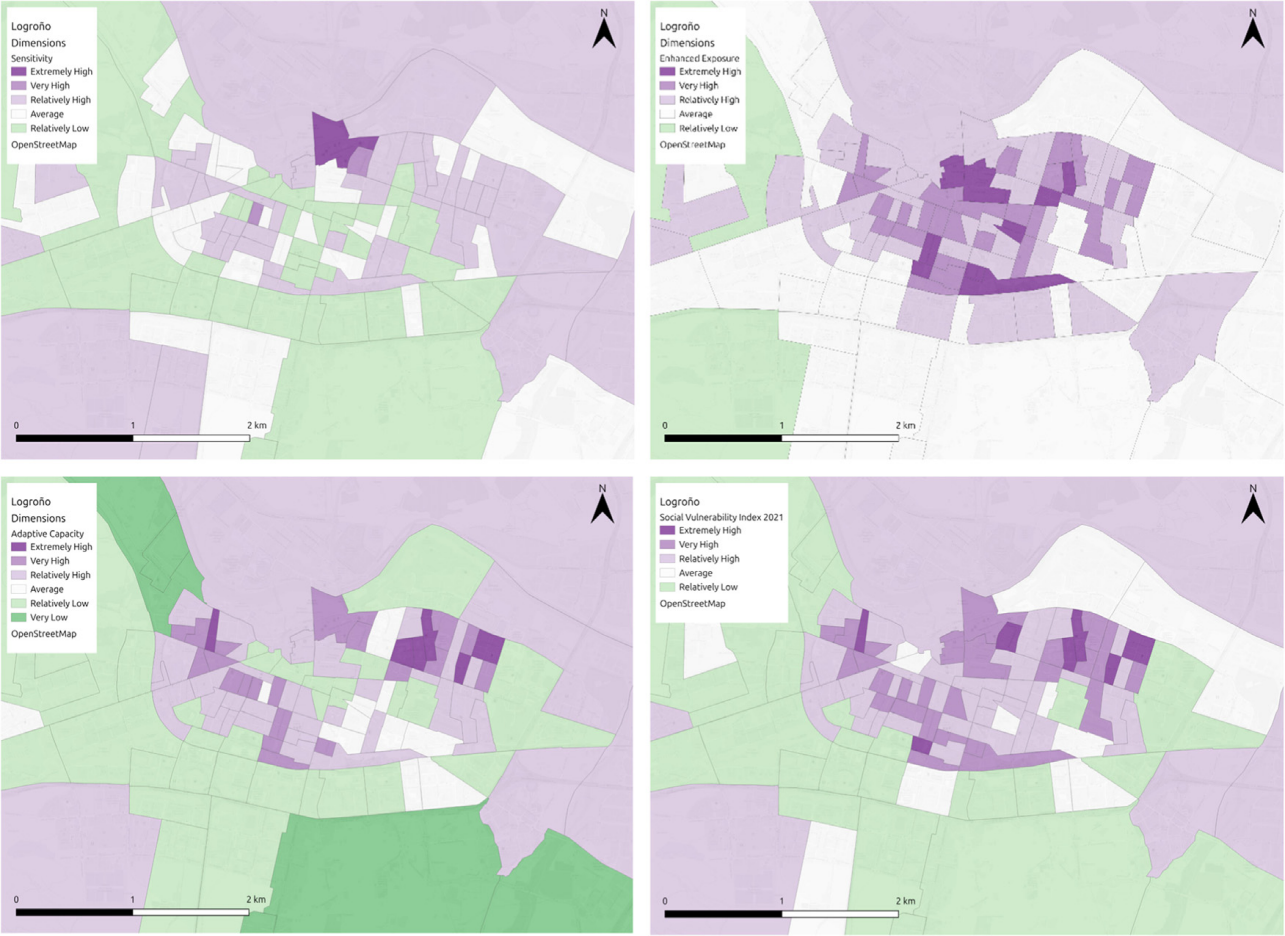
Visual representation of the SVI for heat hazard in Logroño, alongside dimension specific representations (top left: sensitivity; bottom left: adaptive capacity; top right: enhanced exposure; bottom right: overall vulnerability).

*Tier 2*: This second scenario is recommended for cases where domains have single indicators (Fig. 4) or where there is a lack of information related to missing indicators. For these domains the weights are halved. See Eq. 3.

(3) $$SVI=\;\sum_{1}^{n}\sum_{1}^{m}W_{mn}*ind_{m}$$

$$W_{mn}=\;\left\{ \begin{array}{c} \frac{1}{2m}\;if\;m=1\;or\;missing\;key\;indicators\\ \frac{1}{m}\;otherwise \end{array}\right.$$

where SVI is the vulnerability index, *ind_m_* is the m^th^ indicator, m is the total number of indicators for each n domain, and W_mn_ is the weight of the m^th^ indicator for the n^th^ domain.

**Fig. 5:**
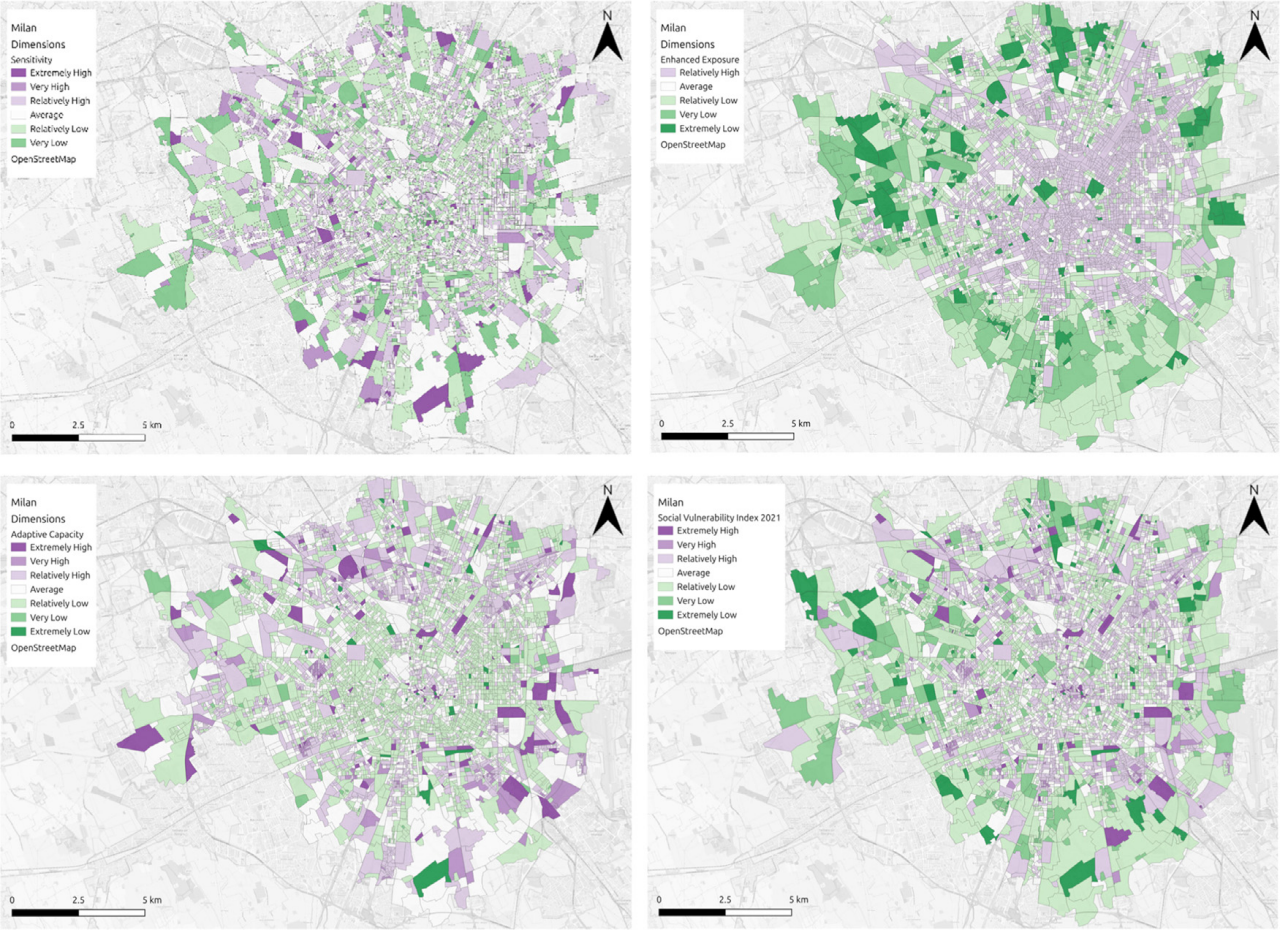
Visual representation of the SVI for heat hazard in Milan, alongside dimension specific representations (top left: sensitivity; bottom left: adaptive capacity; top right: enhanced exposure; bottom right: overall vulnerability).

*Tier 3*: This third scenario is used in cases where there is only a single domain in any of the three main dimensions (Fig. 5). In these cases, the methodology applied to calculating the weighting coefficient is the same as that of the second tier, but the final index is considered less robust and based on a dimension with less information. Some consideration should be taken before making climate decisions based entirely on the social vulnerability index in this scenario.

*Tier 4*: This last option is considering cases where there are dimensions missing. In this case the approach needs to be redefined, and the robustness of the index is highly affected. This approach may be used to estimate vulnerability information related to social and environmental aspects but should not be considered a Social Vulnerability Index.

We propose a statistical approach based only on the indicators available and not considering the domains. To ensure the most robust output possible, effectively capturing the underlying patterns in the data, we propose using Principal Component Analysis (PCA), which is superior to the equal weighting option in this circumstance.

When calculating vulnerability indices, multicollinearity among indicators can lead to biased results, as highly correlated features may disproportionately influence the overall index. Principal Component Analysis (PCA) is an effective dimensionality reduction technique that addresses this issue by transforming a set of correlated indicators into a smaller number of uncorrelated principal components (PCs).

The primary purpose of PCA in calculating vulnerability indices is to reduce multicollinearity by transforming correlated indicators into orthogonal components, thereby eliminating redundancy caused by collinearity. Additionally, PCA simplifies the dataset by identifying the minimum number of components that explain the maximum variance, ensuring the focus remains on the most significant features while discarding noise or less-informative indicators. Furthermore, the contribution of each indicator to the principal components can be utilized to derive weight coefficients, ensuring that the importance of correlated features is appropriately distributed.

To derive weight coefficients for calculating vulnerability indices, the process begins by generating a correlation matrix for all indicators to evaluate their relationships and identify potential multicollinearity. This matrix highlights strong positive or negative correlations, which can result in redundancy. The next step involves performing Principal Component Analysis (PCA) using the correlation matrix as input. PCA transforms the original correlated indicators into uncorrelated principal components, addressing the redundancy and collinearity issues.

Once the PCA is performed, the proportion of total variance explained by each principal component is extracted. These variance proportions indicate the relative importance of each component in representing the dataset's information. To ensure components that explain more variances have a greater influence, the values of each principal component are weighted by its corresponding variance proportion. Finally, a weighted mean approach can be used, where the weight coefficients derived from PCA are applied to calculate the final vulnerability score for each area. This approach ensures all indicators contribute appropriately to the index, avoiding disproportionate influence of highly correlated features while retaining the essential variability of the dataset. PCA has gained significant attention in recent years for constructing socioeconomic status indices. By integrating PCA with appropriate weighting and aggregation methods [[19], [20], [21]] researchers and policymakers can develop more accurate and reliable indices that better inform decision-making processes.

### Aggregation

**Fig. 6:**
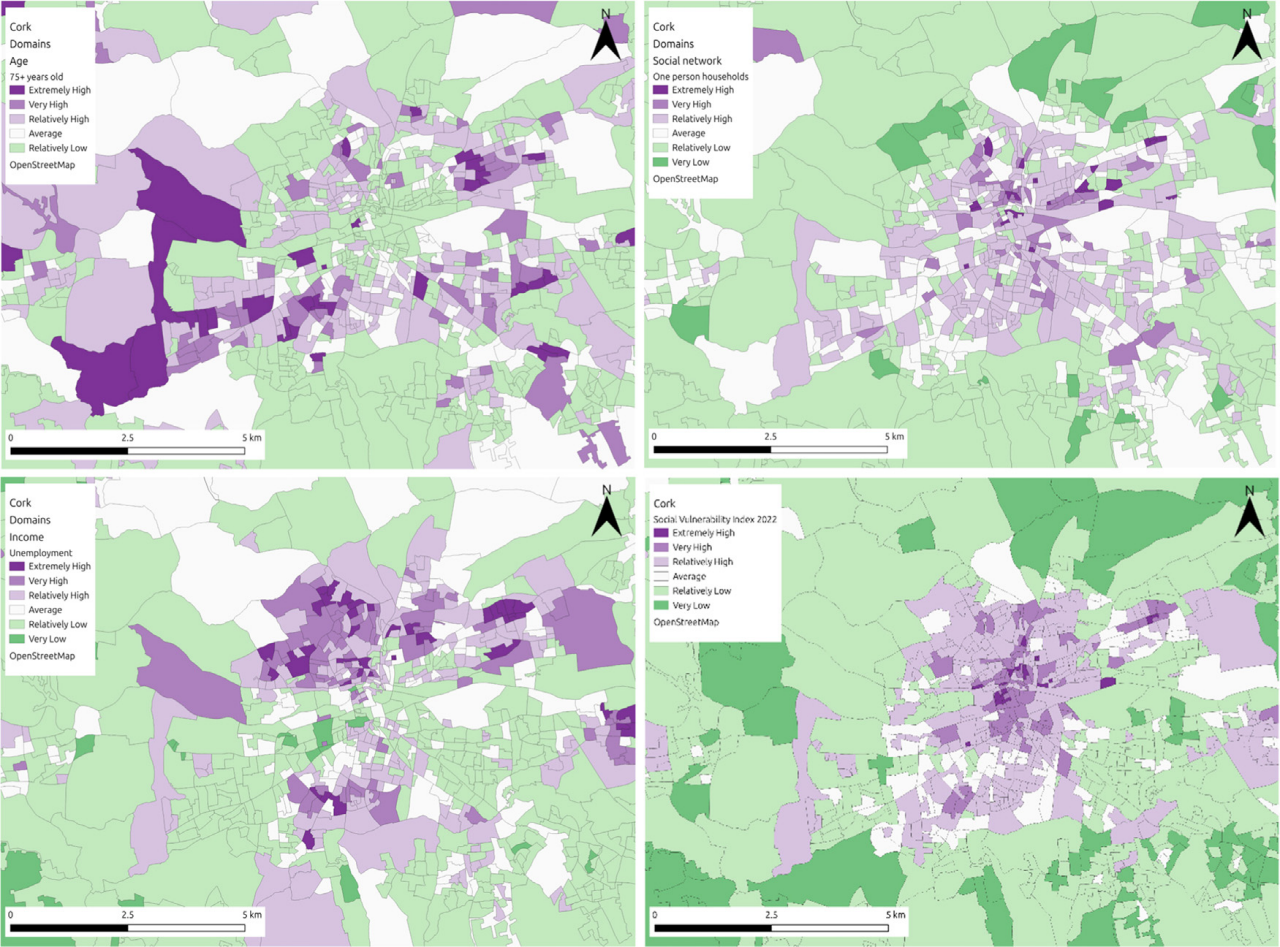
Visual representation of the SVI for flooding in Cork, alongside indicator specific representations (top left: population aged over 75; bottom left: unemployment; top right: one person households; bottom right: overall vulnerability).

While Wehbe and Baroud {19} have highlighted limitations to using composite indicators with regard to identifying specific patterns, so that the needs of specific vulnerable groups can be addressed, the research here attempts to counteract this by providing an option for each of the indicators to be viewed as a single stand-alone product, before being combined into dimensions and then finally into a vulnerability index (Fig. 6).

## Accessibility

To ensure widespread accessibility, all material in this research can be reproduced using the open-sourced code and data. The dataset chosen for socio-economic data was the national census which employs open access baseline data that is familiar to users globally and is consistently collected on a ten-year timeframe in almost all countries, providing an excellent repository of information that exists as a global standard.

All code and data from each of the three case studies in the REACHOUT project (Cork, Logroño, Milan) are available to users in an open repository (Table 4). As the case studies in Rimini and Northern Ireland (outlined in supplementary material) are further developed, these inputs and outputs will also be included in the open repository.

**Table 4:** Information inputs and outputs for the SVI (Data, Code and Guidance).

Location	Input data source	Output data	Code & Guidance
Cork	Irish census data: https://www.cso.ie/en/census/ Copernicus Tree Cover Density: https://doi.org/10.2909/486f77da-d605-423e-93a9-680760ab6791 Copernicus Imperviousness: https://doi.org/10.2909/3e412def-a4e6-4413-98bb-42b571afd15e	EU Open Research Repository, D. Dunne, C. Walther, D. McCullagh, Characterisation of Social Vulnerability to the environmental hazard of flooding in Cork City and County, Ireland, derived from national census and EU Copernicus datasets. (1.0.0) [Data set] (2024c) Zenodo. https://doi.org/10.5281/zenodo.13913850	Jupyter notebooks: https://ucc-climpadap.github.io/SVIBook/Cities/Cork.html
Logroño	Spanish census data: https://www.ine.es/en/ Copernicus Tree Cover Density: https://doi.org/10.2909/486f77da-d605-423e-93a9-680760ab6791 Copernicus Imperviousness: https://doi.org/10.2909/3e412def-a4e6-4413-98bb-42b571afd15e	EU Open Research Repository, D. Dunne, C. Walther, D. McCullagh, Characterisation of Social Vulnerability to the environmental hazard of heat in Logroño, and the surrounding La Rioja region in Spain, derived from national census and EU Copernicus datasets. (1.0.0) [Data set]. Zenodo (2024b) https://doi.org/10.5281/zenodo.13909225	Jupyter notebooks: https://ucc-climpadap.github.io/SVIBook/Cities/Logrono.html
Milan	Italian census data: https://www.istat.it Copernicus Tree Cover Density: https://doi.org/10.2909/486f77da-d605-423e-93a9-680760ab6791 Copernicus Imperviousness: https://doi.org/10.2909/3e412def-a4e6-4413-98bb-42b571afd15e	EU Open Research Repository D. Dunne, C. Walther, D. McCullagh, Characterisation of Social Vulnerability to the environmental hazard of heat in Milan, derived from national census and EU Copernicus datasets (1.0.0) [Data set] (2024a) Zenodo https://doi.org/10.5281/zenodo.13894260	Jupyter notebooks: https://ucc-climpadap.github.io/SVIBook/Cities/Milan.html

To further increase the ease of access and replicability for a variety of users, especially those without abundant (technical and financial) resources available, a series of Juypter notebooks with step-by-step user guidance is available for Cork, Milan and Logroño and is being developed for Rimini and Northern Ireland. The SVI tool has also been included in the EU Mission on Adaptation to Climate Change tools database MIP4ADAPT at [https://climate-adapt.eea.europa.eu/en/mission/solutions/tools/social-vulnerability-index-svi], increasing availability and access for diverse users.

## Future method developments

The authors are currently exploring future technological and accessibility developments to further advance the SVI so that it can be as effective as possible in supporting equity in climate decision-making. This study has highlighted the differences between countries in the level of data collection, collation and resolution in the census datasets. Each of the three focus areas used a different tier of weighting methodology due to data availability at the national level. The authors are aiming to include different local data that may be available at the municipal level to produce additional indicators and make the SVI more robust, while still trying to keep it as accessible as possible. An example of a method being investigated is the use of open street maps to include critical infrastructure, or data that is collected by local authorities, but is still available for users to request, for example the locations of public water sources in a city. Given the timeframe for census data collection in each country is generally every ten years, this inclusion of local level datasets can allow for potentially more current information on socio-economic elements in an area. To support the needs of planning departments within regions, the SVI spatial areas can also be adjusted to match administrative boundaries.

While the SVI can be used as a stand-alone tool, it has also been integrated into both the FloodAdapt and Thermal Assessment tools in the REACHOUT project. This has allowed cities to use tailored flooding and heat hazard information alongside socio-economic information while planning for climate change. In Ireland and Northern Ireland, indicators that include projected future climate risks for rainfall and extreme heat are also being incorporated into the enhanced exposure dimension, with the caveat that the socio-economic information is based on current data. This will allow decision-makers to understand the risk that their cities and regions may face in the future, and how some populations may be even more susceptible than they currently are. In the future the SVI will also be tailored to include indicators that address additional hazard indicators such as those for wildfire and drought, to understand a range of climate risks.

While this study outlines a number of different tiered weighting methods, dependent on data availability and technical expertise, the authors aim to apply and test a combination of approaches in a single area to allow for comparison. This will also include an additional approach, using expert knowledge in a participatory approach through a modified Delphi method as outlined by Rufat et al. [21]. The intention of this work is to generate a more robust SVI final vulnerability score that incorporates both quantitative and qualitative methodologies.

## Method results and validation

**Fig. 7:**
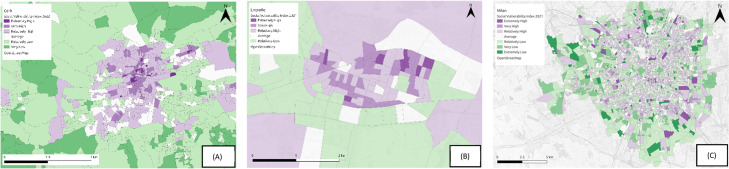
SVI maps of (A) Cork, (B) Logroño and (C) Milan.

The figure below (Fig. 7) highlights the social vulnerability index map outputs to flooding (Cork) and heat hazard (Logroño and Milan) in each of the three regions, using the updated methodology outlined in this article. In Cork (Fig. 7a) the vulnerability ranges from extremely high to very low, with a clear distinction between the city centre area and the outskits of the city. Areas in the city centre are the most vulnerable in the city, with the outskirts much less vulnerable to flooding. In Logroño (Fig. 7b), the vulnerability ranges from extremely high to relatively low, with areas in the centre, the north and east of the municipality scoring much higher for vulnerability to heat, than the areas to the south and west. In Milan (Fig. 7c) the vulnerability ranges between extremely high and extremely low, with higher areas of vulnerability primarily located in the city centre and areas of lower vulnerability concentrated towards the outskirts of the city. In both the north and southeast of the city, areas of both extremely high and low vulnerability exist side by side, while these differences are visible to some degree in other areas of the city, it is more pronounced in these regions. In Cork and Milan the resolution of the data is quite high and differences in SVI scores are obvious over small spatial areas, making the SVI a useful tool for engaging the public. In Logroño, the data is at a much lower resolution, something which may be disguising smaller pockets of vulnerability within the municipality.

In order to validate the indicator selection for the methodology, the authors carried out a broad and in-depth literature review on social vulnerability indexes and the different factors that can impact vulnerability to specific climate hazards. However, the primary function of the development of the SVI was its usability for decision-makers, in particular those in local government who will be implementing climate adaptation measures. The work involved extensive discussions with local authorities in each of the case study areas over a three-year period, to determine data availability and to develop a roadmap for how the SVI could be best utilised within their day-to-day operations. Additionally, the final visual outputs were also sense checked with local authorities to identify any outputs that conflicted with their local knowledge.

**Fig. 8:**
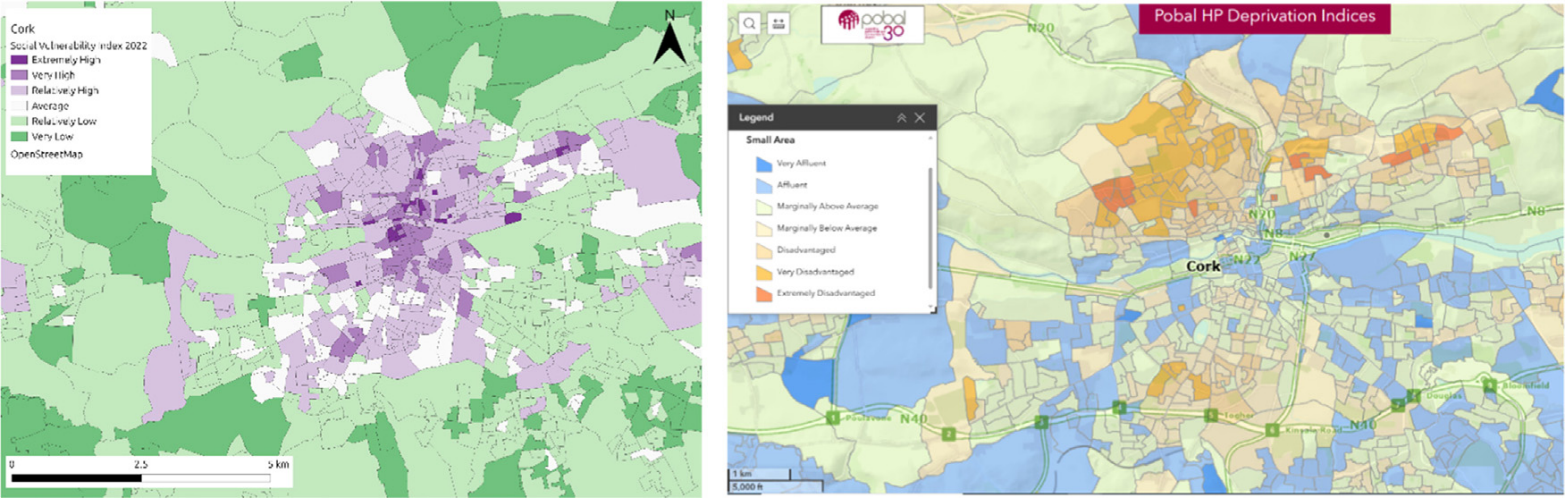
SVI overall vulnerability map for Cork City (left), beside the Pobal Deprivation Index for Cork City (right) (accessible at: https://data.pobal.ie/portal/apps/sites/#/pobal-maps).

As census information is generally considered to be the ‘pre-eminent approach’ of collecting demographic information and often serves as the ‘benchmark for the national statistical system’ [22] it is frequently used for the validation of other datasets. As this study uses census data to provide information, the method cannot be validated against it, however, a certain level of data quality is assumed based on the high quality of national census datasets. In an effort to further validate the SVI, it was compared against the Pobal Deprivation Index for the city of Cork. Similar validation tools were unavailable for Logroño and Milan. The Pobal Deprivation Index for Ireland utilises three components (demographic profile, social class and labour market situation) and includes similar indicators to the SVI [23]. It is therefore able to be compared, to some degree, with the SVI in Cork (Fig. 8). There is a good deal of overlap in the overall vulnerability levels in the north of the city, with differences primarily between the SVI suggesting very high level of vulnerability and the Pobal Index suggesting that these areas are extremely disadvantaged, so the degree of vulnerability/disadvantage. In the east and west of the city there is again general agreement between the maps that these areas are less vulnerable and more affluent. There are some minor differences in the south of the city but once again there is general alignment that these areas are more average in their vulnerability and disadvantage. The key differences in the maps are found in the city centre, and in particular, along the river, where the Pobal Deprivation Index lists these areas as very affluent, while the SVI shows them to be high or extremely high vulnerability. To some degree this further supports the SVI map of Cork as the indicators chosen where based on vulnerability to flooding, while the Pobal maps are based on deprivation indicators only. The proximity to the river, the main source of flooding in the city, is a key factor influencing the SVI maps in Cork. The Pobal Deprivation Index has aggregated its indicators and into the 3 components and therefore a more detailed analysis cannot be carried out.

## Limitations

SVI indicators, obtained from datasets containing socio-economic information, are based on extensive research including a review of existing literature and the interpretation of factors affecting social vulnerability. Interpretation of some of these indicators are contestable and open to debate as they present a proxy for specific information that is unavailable. For example, not all individuals who have high commute times to work or school will necessarily have lower physical access to critical services, however, it is much more likely. These limitations are difficult to avoid given the protected nature of individual personal information in many countries and the necessity to use proxies. However, efforts should be made to avoid indicators which are highly contentious.

The SVI method we have developed introduces the option of tiered methodologies for levels of robustness, this is to compensate for cases in which very few indicators are available to create the SVI. However, even with this option, case studies utilising the tier 4 method are not robust enough to establish an SVI score that should be utilised for decision-making in an area, unless additional and complementary information is available. Additionally, the enhanced exposure dimension, utilises only proxy information for both flooding and heat hazard. This is less robust than if direct climate data is applied showing the extent or severity of flooding or heatwaves. While this is mitigated to an extent by integrating the SVI with additional tools, it is currently a limitation in the SVI.

As the SVI produces a relative ranking of vulnerability, the score is only valid at the scale at which the analysis was done. For example, this means that the SVI scores for Milan and Rimini cannot be compared although they are both taken from Italian census data and use the same indicators. In the case of Ireland and Northern Ireland the analysis has been carried out at the national scale and therefore all regions in these two countries can be compared. In the case of Logroño, the wider region of La Rioja was included in the mapping so that areas within this region could be compared.

Finally, the method by which a large proportion of socio-economic datasets are validated, is through comparison with the national census. As previously mentioned in the validation section, it is the census datasets that have been used to create the SVI in all areas and therefore validation against it is not possible in this case. While the datasets used are robust and all outputs have been sense checked with local authorities in each of the regions, further work should strive to include a variety of methods for comparison, and to complete ground truthing through detailed and comprehensive surveys in the localities where the SVI has been used to ensure the most accurate representation of social vulnerability to climate hazards is produced and validated in each region.

## Ethics statements

Ethical approval was not sought for the present study because the work did not involve human subjects or data that was not freely available. All ethical guidelines outlined by both the journal and the research institution (University College Cork) have been followed.

## CRediT authorship contribution statement

**Denise McCullagh**: Conceptualization, Formal analysis, Investigation, Methodology, Validation, Visualization, Writing - original draft; **Walther Cámaro-García**: Data curation, Formal analysis, Investigation, Methodology, Software, Validation, Visualizatio, Writing - original draft; **Declan Dunne:** Data curation, Formal analysis, Investigation, Methodology, Software, Validation, Visualization, Writing - review & editing; **Parvaneh Nowbakht**: Formal analysis, Investigation, Methodology, Software, Validation, Visualization, Writing - review & editing; **Lydia Cumiskey**: Writing - review & editing; **Cathal Gannon**: Investigation; **Christopher Phillips**: Writing - review & editing.

## Declaration of competing interest

The authors declare that they have no known competing financial interests or personal relationships that could have appeared to influence the work reported in this paper.

## Data Availability

Data will be made available on request.

## Appendix Supplementary materials

Image, application 1

## References

[1] Fitton J.M., O'Dwyer B., Maher B. (2021). Developing a social vulnerability to environmental hazards index to inform climate action in Ireland. Irish Geogr..

[2] Clarke B., Otto F., Stuart-Smith R., Harrington L. (2022). Extreme weather impacts of climate change: an attribution perspective. Environ. Res. Climate.

[3] Bouwer L.M. (2019). Observed and projected impacts from extreme weather events: implications for loss and damage. Loss Damage Clim. change: Concepts, Methods And Policy Options.

[4] Newman R., Noy I. (2023). The global costs of extreme weather that are attributable to climate change. Nat. Commun..

[5] Spielman S.E., Tuccillo J., Folch D.C., Schweikert A., Davies R., Wood N., Tate E. (2020). Evaluating social vulnerability indicators: criteria and their application to the Social Vulnerability Index. Natural hazards.

[6] Klinsky S., Mavrogianni A. (2020). Climate justice and the built environment. Buildings and Cities.

[7] Siagian T.H., Purhadi P., Suhartono S., Ritonga H. (2014). Social vulnerability to natural hazards in Indonesia: Driving factors and policy implications. Natural hazards.

[8] Mavhura E., Manyena B., Collins A.E. (2017). An approach for measuring social vulnerability in context: The case of flood hazards in Muzarabani district. Geoforum..

[9] A. Kazmierczak, G. Cavan, A. Connelly, S. Lindley, Mapping flood disadvantage in Scotland 2015 (2015) Edinburgh: Scottish Government.

[10] S. Lindley, J. O'Neill, Flood disadvantage in Scotland: mapping the potential losses in well-being. Scottish Government Social Research, Edinburgh (2013).

[11] S. Lindley, J. O'Neill, J. Kandeh, N. Lawson, R. Christian, M. O'Neill, Climate change, justice and vulnerability. Joseph Rowntree Foundation, York, (2011) 1-177.

[12] Cutter S.L., Emrich C.T., Webb J.J., Morath D. (2009). Social vulnerability to climate variability hazards: A review of the literature. Final Report to Oxfam America.

[13] Dong J., Peng J., He X., Corcoran J., Qiu S., Wang X. (2020). Heatwave-induced human health risk assessment in megacities based on heat stress-social vulnerability-human exposure framework. Landsc. Urban. Plan..

[14] Fatemi F., Ardalan A., Aguirre B., Mansouri N., Mohammadfam I. (2017). Social vulnerability indicators in disasters: Findings from a systematic review. International journal of disaster risk reduction.

[15] Inostroza L., Palme M., De La Barrera F. (2016). A heat vulnerability index: spatial patterns of exposure, sensitivity and adaptive capacity for Santiago de Chile. PLoS. One.

[16] Kumar G.D., Pradhan K.C. (2024 a). Assessing the district-level flood vulnerability in Bihar, eastern India: an integrated socioeconomic and environmental approach. Environ. Monit. Assess..

[17] Moreira L.L., de Brito M.M., Kobiyama M. (2021). A systematic review and future prospects of flood vulnerability indices. Natural Hazards and Earth System Sciences Discussions.

[18] Oulahen G., Shrubsole D., McBean G. (2015). Determinants of residential vulnerability to flood hazards in Metro Vancouver. Canada. Natural Hazards.

[19] Cutter S.L (2024). The Origin and Diffusion of the Social Vulnerability Index (SoVI). International Journal of Disaster Risk Reduction.

[20] Isia I., Hadibarata T., Hapsari R.I., Jusoh M.N.H., Bhattacharjya R.K., Shahedan N.F. (2023). Assessing social vulnerability to flood hazards: a case study of Sarawak's divisions. International journal of disaster risk reduction.

[21] Rufat S., Tate E., Emrich C.T., Antolini F. (2019). How valid are social vulnerability models?. Ann. Am. Assoc. Geogr..

[22] Inostroza L., Palme M., De La Barrera F. (2016). A heat vulnerability index: spatial patterns of exposure, sensitivity and adaptive capacity for Santiago de Chile. PLoS. One.

[23] Baffour B., Valente P. (2012). An evaluation of census quality. Stat. J. IAOS..

