# Supplementary material for: Development of a social vulnerability index: Enhancing approaches to support climate justice
Source: MethodsX. 2025 Mar 26;14:103290. doi: 10.1016/j.mex.2025.103290 (PMC11999302; doi:10.1016/j.mex.2025.103290)
Supplement: Supplementary file 1 [file mmc1.docx]

**Supplementary material *and/or* additional information**

**Indicators (Used and Potential Options)**

The indicators outlined in the text above (Tables 1 - 3) are those used in our study, however, there are a wide range of socio-economic and environmental indicators that can be used if there is data available. While not an exhaustive list, the indicators outlined below (Tables 5 -10) provide a broad range of potential information that may be available to local and regional authorities for developing SVI indicators for heat and flood.

*Table 5: Sensitivity indicators for Flooding Hazards*

| DOMAIN | FLOOD INDICATOR | RATIONALE | REFERENCE |
| --- | --- | --- | --- |
| Age | Nursing home residents per capita | Decreased mobility; even when healthy often have a limited fixed income  The elderly are at greater risk of adverse health outcomes with elevated hospitalization and mortality rates especially during extreme events, probably due to excess strain exerted on pre-existing morbidities.  Physiologically, the elderly have a greater susceptibility to the effects of a climate hazard and potentially have other chronic health issues. | [8, 11, 12, 13, 14, 15, 16, 19, 20, 21] |
|  | Population 65 Years and Older or Disabled |  |  |
|  | Elderly: Proportion of households that entirely consist of pensioners |  |  |
|  | % people over 75 years old [or similar range] |  |  |
|  | Median age of the population |  |  |
|  | % people under 5 years old [or similar range] | Physiologically, the young have a greater susceptibility to the effects of a climate hazard.  Population with higher percentage of children have lower coping capacity and therefore increased vulnerability  Extremes along the age spectrum affect mobility out of harm’s way and increase the burden of care following a damaging event. This is partially the result of reductions in services that may make recovery especially difficult for age dependent populations  Population with higher percentage of children have lower coping capacity and therefore increased vulnerability  Dependents under law until 18 and are less mobile | [2, 3, 8, 10, 11, 12, 13, 14, 15, 16, 21, 22, 23, 24, 25] |
|  | Population aged under 15 years |  |  |
|  | Population Under 18 Years |  |  |
| Health | Poor health: Proportion of adult population with poor health | Physiologically, those in poor physical health may struggle if short term evacuation was required due to mobility and health complications. Adapting to a new living situation and moving away from a community support network who they may be reliant upon may negatively impact those of poor health. | [1, 3, 10, 21, 26, 27, 28] |
|  | % people whose day-to-day activities are limited (by health) |  |  |
|  | Population having difficulty in seeing, hearing, walking, using stairs, using hands or fingers or doing other physical activities, learning, remembering/ concentrating, emotional, psychological /mental, other health problems/long-term conditions for six months and above |  |  |
|  | % households with at least one person with long-term limiting illness |  |  |
|  | % of Incapacity Benefit/Severe Disablement Allowance claimants |  |  |
|  | % people living in medical and care establishments |  |  |
|  | Preexisting health conditions |  |  |
|  | Population with activity limitations due to emotional, psychological or mental health conditions | Adapting to a new living situation and moving away from a community support network who they may be reliant upon may negatively impact those of poor health. | [22, 26, 29, 30] |
|  | % population with poor mental health |  |  |

*Table 6: Sensitivity indicators for Extreme Heat Hazards*

| DOMAIN | FLOOD INDICATOR | RATIONALE | REFERENCE |
| --- | --- | --- | --- |
| Age | Nursing home residents per capita | Decreased mobility; even when healthy often have a limited fixed income  The elderly are at greater risk of adverse heat-related health outcomes with elevated hospitalization and mortality rates especially during EH events in the summer, probably due to excess strain exerted on pre-existing morbidities.  Physiologically, the elderly have a greater susceptibility to the effects of a climate hazard and potentially have other chronic health issues. | [6, 11, 13, 16, 17, 18, 19] |
|  | Population 65 Years and Older or Disabled |  |  |
|  | Elderly: Proportion of households that entirely consist of pensioners |  |  |
|  | % people over 75 years old [or similar range] |  |  |
|  | Median age of the population |  |  |
|  | % people under 5 years old [or similar range] | Physiologically, the young have a greater susceptibility to the effects of a climate hazard.  Population with higher percentage of children have lower coping capacity and therefore increased vulnerability  Extremes along the age spectrum affect mobility out of harm’s way and increase the burden of care following a damaging event. This is partially the result of reductions in services that may make recovery especially difficult for age dependent populations  Population with higher percentage of children have lower coping capacity and therefore increased vulnerability  Dependents under law until 18 and are less mobile | [3, 6, 9, 13, 16, 18, 22, 23, 31] |
|  | Population aged under 15 years |  |  |
|  | Population Under 18 Years |  |  |
| Health | Poor health: Proportion of adult population with poor health | Physiologically, those in poor physical health may struggle if short term evacuation was required due to mobility and health complications. Adapting to a new living situation and moving away from a community support network who they may be reliant upon may negatively impact those of poor health. | [1, 3, 18, 21, 23, 26, 28, 31] |
|  | % people whose day-to-day activities are limited (by health) |  |  |
|  | Population having difficulty in seeing, hearing, walking, using stairs, using hands or fingers or doing other physical activities, learning, remembering/concentrating, emotional, psychological/mental, other health problems/long-term conditions for six months and above |  |  |
|  | % households with at least one person with long-term limiting illness |  |  |
|  | % of Incapacity Benefit/Severe Disablement Allowance claimants |  |  |
|  | % people living in medical and care establishments |  |  |
|  | Preexisting health conditions |  |  |
|  | Population with activity limitations due to emotional, psychological or mental health conditions | Exposure to extreme heat events can have a substantial negative impact on individuals with mental illness. | [30] |
|  | % population with poor mental health |  |  |
| Gender | Female Population | Heat-vulnerability studies have found women at a higher risk of heat-related mortality and morbidity than men regardless of age group | [18] |

*Table 7: Adaptive Capacity indicators for Flooding Hazards*

| DOMAIN | INDICATOR | RATIONALE | REFERENCE |
| --- | --- | --- | --- |
| Income | Per Capita Income (€) | Wealth enables the ability to prepare, respond to and recover from losses due to institutional resilience such as insurance, social safety nets, and entitlements. | [8] |
|  | Net household income: Proportion of households with income of less than the national average per week | Those on a low income, without earning capacity or in financial difficulty could easily be pushed into further problems, limiting their adaptive capacity | [1, 8, 11, 12, 13, 14, 15, 16, 18, 20] |
|  | No savings: Proportion of adult population with no savings |  |  |
|  | Number of Income Support claimants |  |  |
|  | Unemployment |  |  |
|  | Number of families receiving tax credits |  |  |
|  | Population with a shelter-cost-to-income ratio of over 30% |  |  |
|  | Population with low-income status based on national level (prevalence of low income) |  |  |
|  | Median per capita home value (owner-estimated) as a proxy for per capita wealth |  |  |
|  | Median per capita income of census family for all persons aged 15 (or similar) or older |  |  |
|  | Secured or unsecured loans: Proportion of adult population with secured or unsecured loans |  |  |
|  | Percent households receiving social security benefits |  |  |
|  | People Below Poverty line in Past 12 Months |  |  |
|  | Backward / deprived population (%) |  |  |
|  | Population Below Poverty line (%) |  |  |
|  | Number of Pension Credit claimants |  |  |
|  | Population aged 20 to 64 below poverty line (%) |  |  |
|  | Dependents: Children under 18 years live with single parent only in families and subfamilies (%) | One parent households are more  likely to have less disposable income  compared to 2 parent households | [24, 33] |
|  | Dependents: Percent children living in 2 parent families |  |  |
|  | Families with 3 or more dependants | Households with 3 or more dependants are more likely to have less disposable income compared to other households due to social, economic and family care responsibilities | [8, 22,23] |
| Information access/ use  Local knowledge | % of households with no internet | People in households with no internet  may find it difficult to find information,  pre, during, and post event | [1] |
|  | Education: Proportion of adult population who left school before or at 16 | Those with lower education attainment hinders the ability to understand, interpret and act on warning information. | [1,32, 34, 35] |
|  | Education: Population aged 18 or older (or similar) with no certificate/diploma/degree |  |  |
|  | Education: Illiterate population (%) |  |  |
|  | Population with no knowledge of the official language | Communication difficulties due to language barriers. | [8, 14, 19] |
|  | Population who do not speak English well or at all |  |  |
|  | Percentage of foreign nationals | Foreign nationals and those who are new to the area are likely to have less local knowledge and be less aware of hazards and how to act upon warning information received. | [3, 23] |
|  | Those who have lived in the area less than 1 year |  |  |
|  | % new addresses located in flood risk areas | People living in areas with a high turnover of population may be less aware of the likelihood of being affected by floods, how to respond and where to seek support | [3] |
| Mobility | Dependent population (%) | A high dependant population may make short-term evacuation of people and possessions more difficult | [23] |
|  | Households with no motor car | Without a car, short-term evacuation of people and possessions is more difficult. Additionally, if a person is repatriated to a new location, without a car travelling between a workplace and school without a car may be problematic | [1] |
| Physical access | Proximity/Access to Hospitals | Those with easy access to health care are more likely to be able to respond to and physical injury/illness during flooding events. Physiologically, those in poor physical health may struggle if short term evacuation was required due to mobility and health complications. Adapting to a new living situation and moving away from a community support network who they may be reliant upon may negatively impact those of poor health. | [27, 28] |
|  | Proximity to Schools | School buildings are likely to be used as rescue sites during emergencies. | [28] |
|  | Public/Community Centres | Public centres are likely to be used as rescue sites during emergencies | [28] |
|  | Residential Density | Higher population density presents evacuation difficulties | [5, 8, 36, 37] |
|  | % of people with >1 hour to  work/school | People with long commutes to work/  school are likely living in areas with low  service provision | [1] |
|  | Access to treated (public) water supply | Those without access to treated water, such as through a private water supply may not have as sophisticated water quality monitoring as compared to the public supply making the occurrence of water borne diseases and illness more likely after a flooding event | [1] |
| Social network | Households with one person | One-person households are more likely  to have socially isolated people and  potential for a reduced social network | [1, 4] |
|  | Volunteering | Areas with higher proportions of the population volunteering are likely to  have more social ties and a stronger social network due to participation in these (often community based) activities | [38] |
|  | % children of primary school age | Areas with higher proportions of  primary school children are likely to  have more social ties due to parents  participating in school-related activities | [1, 9] |
|  | Number of domestic break-ins | Fear of crime and the anxiety that this could indicate that residents are away may prevent deployment of cautionary measures such as door guards.  Areas of high crime have been shown to have lower community ties and weaker social networks, which can impact recovery after a climate hazard | [3, 39, 40, 41] |
|  | Unoccupied dwellings | A high level of vacancy in an area can indicate higher levels of crime and social disorder | [42, 43] |
| Tenure | Households renting | Those in rented accommodation have a  reduced ability to adapt their homes. | [1, 9] |

*Table 8: Adaptive Capacity indicators for Extreme Heat Hazards*

| DOMAIN | INDICATOR | RATIONALE | REFERENCE |
| --- | --- | --- | --- |
| Income | Per Capita Income (€) | Wealth enables the ability to prepare, respond to and recover from losses due to institutional resilience such as insurance, social safety nets, and entitlements. | [1, 3, 5, 8, 11, 13, 36] |
|  | Net household income: Proportion of households with income of less than the national average per week | Those on a low income, without earning capacity or in financial difficulty could easily be pushed into further problems, limiting their adaptive capacity. |  |
|  | No savings: Proportion of adult population with no savings |  |  |
|  | Number of Income Support claimants |  |  |
|  | Unemployment |  |  |
|  | Number of families receiving tax credits |  |  |
|  | Population with a shelter-cost-to-income ratio of over 30% |  |  |
|  | Population with low-income status based on national level (prevalence of low income) |  |  |
|  | Median per capita home value (owner-estimated) as a proxy for per capita wealth |  |  |
|  | Median per capita income of census family for all persons aged 15 (or similar) or older |  |  |
|  | Secured or unsecured loans: Proportion of adult population with secured or unsecured loans |  |  |
|  | Percent households receiving social security benefits |  |  |
|  | People Below Poverty line in Past 12 Months |  |  |
|  | Backward / deprived population (%) |  |  |
|  | Population Below Poverty line (%) |  |  |
|  | Number of Pension Credit claimants | The elderly are at greater risk of adverse heat-related health outcomes with elevated hospitalization and mortality rates especially during extreme heat events in the summer, probably due to excess strain exerted on pre-existing morbidities. |  |
|  | Population aged 20 to 64 below poverty line (%) |  |  |
|  | Dependents: Children under 18 years live with single parent only in families and subfamilies (%) | One parent households are more  likely to have less disposable income  compared to 2 parent households | [24, 33] |
|  | Dependents: Percent children living in 2 parent families |  |  |
|  | Families with 3 or more dependants | Households with 3 or more dependants are more likely to have less disposable income compared to other households due to social, economic and family care responsibilities | [22, 26, 29] |
| Information access/ use  Local knowledge | % of households with no internet | People in households with no internet  may find it difficult to find information,  pre, during, and post event | [1] |
|  | Education: Proportion of adult population who left school before or at 16 | Those with lower education attainment hinders the ability to understand, interpret and act on warning information. | [1, 32, 35] |
|  | Education: Population aged 18 or older (or similar) with no certificate/diploma/degree |  |  |
|  | Education: Illiterate population (%) |  |  |
|  | Population with no knowledge of the official language | Communication difficulties due to language barriers. | [8, 19, 44] |
|  | Population who do not speak English well or at all |  |  |
|  | Percentage of foreign nationals | Foreign nationals and those who are new to the area are likely to have less local knowledge and be less aware of hazards and how to act upon warning information received. | [3, 23, 45] |
|  | Those who have lived in the area less than 1 year |  |  |
|  | Experience of previous extreme heat events | Experience of previous heat events can allow for increased preparedness during future events | [49] |
| Mobility | Dependent population (%) | A high dependant population may make short-term evacuation of people and possessions more difficult | [23] |
|  | Households with no motor car | Without a car, short-term evacuation of people and possessions is more difficult. | [1] |
| Physical access | Proximity to Medical Centres | Those with easy access to health care are more likely to be able to respond to physical injury/illness during heatwave events. Physiologically, those in poor physical health may struggle if short term evacuation was required due to mobility and health complications. Adapting to a new living situation and moving away from a community support network who they may be reliant upon may negatively impact those of poor health. | [69] |
|  | Proximity to Schools | Public buildings such as schools and community centres are often used as cooling centres during emergencies. Access to cool spaces allows for increased comfort and adequate restoration of body temperature after heat exposure. | [50] |
|  | Public Centres |  |  |
|  | Residential Density | Higher population density presents evacuation difficulties in an emergency | [5, 8, 36, 37] |
|  | % of people with >1 hour to  work/school | People with long commutes to work/  school are likely living in areas with low  service provision | [1] |
|  | Access to water supply | Those without access to a secure water supply are more likely to have a greater susceptibility to the effects of heat hazard | [51] |
| Social network | Households with one person | One-person households are more likely  to have socially isolated people and  potential for a reduced social network | [1, 4, 52, 53] |
|  | Volunteering | Areas with higher proportions of the population volunteering are likely to  have more social ties and a stronger social network due to participation in these (often community based) activities | [38] |
|  | % children of primary school age | Areas with higher proportions of  primary school children are likely to  have more social ties due to parents  participating in school-related activities | [1, 9] |
|  | Unoccupied dwellings | A high level of vacancy in an area can indicate higher levels of crime and social disorder | [42, 43] |
| Tenure | Households renting | Renters have less ability or incentive to take mitigation action because they do not own the property; renters are usually either transient or do not have financial resources for ownership; might not have adequate insurance; no equity in property | [1, 9] |

*Table 9: Enhanced Exposure for Flooding Hazards*

| DOMAIN | INDICATOR | RATIONALE | REFERENCE |
| --- | --- | --- | --- |
| Physical Environment | Mean value of imperviousness of the new built-up areas [%] | Areas with greater amount of  impervious surface may be more prone  to flood events due to runoff. | [1, 3, 7, 46] |
|  | % Tree cover | Areas with higher tree cover are less prone to runoff and flooding |  |
|  | Land Use/Cover | Areas with higher green cover are less prone to runoff and flooding |  |
|  | FEMA 100-year Flooding Area (%) | Residential and critical infrastructure that are within a known flood zone are at greater risk during a flood event | [3, 21, 56] |
|  | Roadway in Flooding Area (%) |  |  |
|  | Buildings in Flooding Area (%) |  |  |
|  | Critical Facilities in Flooding Area |  |  |
|  | Average No. of Severe Floods in the past 10 years | Previous experience often leads to strong structural and institutional flood defenses associated with reduced flood awareness and self-protective behavior | [8, 57, 58] |
|  | Average No. of death/injury due to floods in the past 10 years |  |  |
| Housing Characteristics | Unoccupied dwellings | Unoccupied dwellings indicate that residents are away and may prevent deployment of cautionary measures such as door guards or sandbags during a flooding event. | [1] |
|  | Percentage of population in informal housing | Informal housing may not offer as much protection to residents as compared to formal housing as it is generally located in more disaster-prone areas | [54] |
|  | Households that are caravans/mobile homes | Caravans/mobile home housing may not offer as much protection to residents as compared to permanent housing | [1, 9] |
|  | Dwelling Construction Year | Households built prior to 1970 may be of poor quality and may not offer as much protection to residents as compared to modern housing |  |
|  | % households with the lowest floor level: ground floor | Houses with the lowest floor at or below ground level are more exposed than dwellings located on higher floors, and occupants and their belongings may be more significantly affected by a flood event | [3, 55] |
|  | % households with the lowest floor level: basement or semi basement |  |  |

*Table 10: Enhanced Exposure for Extreme Heat Hazards*

| DOMAIN | INDICATOR | RATIONALE | REFERENCE |
| --- | --- | --- | --- |
| Physical Environment | Mean value of imperviousness of the new built-up areas [%] | Impervious surfaces have been shown to be strongly related to land surface temperature and the detrimental health effects of the urban heat island | [48] |
|  | % Tree cover | Access to cool spaces allows for increased comfort and adequate restoration of body temperature after heat exposure | [59, 60] |
|  | Land Use/Cover | Areas with higher green cover are less prone to the urban heat island effect | [44, 61] |
|  | Consecutive hot days with temperature max > 30°C and temperature min > 22°C | Continual exposure to extreme temperatures can cause significant heat stress | [62] |
|  | Number of hot days (the days with the maximum temperature higher than a specific value) |  |  |
|  | Number of hours with the heat index in the “danger” or “extreme danger” range | Extreme temperature exposure can cause significant heat stress | [48,51, 62] |
|  | Max Air Temperature |  |  |
|  | Land Surface Temperatures |  |  |
|  | Critical Facilities in Areas of extreme Heat | Extreme temperatures can negatively impact critical infrastructure, in particular the efficient generation of energy can be compromised and transport infrastructure damaged | [63, 64, 65, 66] |
| Housing Characteristics | Air conditioning prevalence | Residents who lack air conditioning of any kind, especially those who lack central air conditioning, are at an increased risk of heat related death | [59, 67] |
|  | Dwelling Construction Year | Older buildings are harder to keep at an appropriate temperature | [45, 48] |
|  | Building Materials | Different building materials retain heat in different levels. Concrete and asphalt used in urban settings and buildings retain heat and take longer to cool down, creating urban heat islands (UHIs) that are substantially warmer than surrounding suburban and rural areas | [3, 47, 60, 68] |

The tables below show the different levels of data availability in Northern Ireland and in Rimini, Italy, and the rationale behind specific indicator selection (Tables11 & 12). In Northern Ireland the Tier 1 weighting methodology is being used to create the SVI, while in the case of Rimini, the Tier 3 weighting methodology is being used.

*Table 11: Indicators used in Northern Ireland to create an SVI*

| **Indicator** | **Domain** | **Dimension** | **Rationale** |
| --- | --- | --- | --- |
| Boys under 5 years of age | Age | Sensitivity | Physiologically, the young have a greater susceptibility to the effects of flooding. |
| Girls under 5 years of age | Age | Sensitivity | Physiologically, the young have a greater susceptibility to the effects of flooding. |
| Males over 75 years of age | Age | Sensitivity | Physiologically, older people have a greater susceptibility to the effects of flooding. |
| Females over 75 years of age | Age | Sensitivity | Physiologically, older people have a greater susceptibility to the effects of flooding. |
| Persons with poor health | Health | Sensitivity | Physiologically, those with an illness have a greater susceptibility to the effects of flooding. |
| Persons with a long-tern health Problem or disability | Health | Sensitivity | Physiologically, those with an illness or disability have a greater susceptibility to the effects of flooding. |
| People with a disability preventing work | Health | Sensitivity | Physiologically, those with a disability have a greater susceptibility to the effects of flooding. |
| Persons with a disability requiring a wheelchair | Health | Sensitivity | Physiologically, those with a disability requiring a wheelchair have a greater susceptibility to the effects of flooding. |
| Persons with a long-term emotional, psychological or mental health condition | Health | Sensitivity | Physiologically, those with mental health conditions may struggle if short term evacuation was required. Adapting to a new living situation and moving away from a community support network who they may be reliant upon may negatively impact them. |
| One parent households | Income | Adaptive Capacity – Ability to Prepare/Respond/Recover | One parent households are more  likely to have less disposable income  compared to other households |
| Households with 3 or more dependent children per family | Income | Adaptive Capacity – Ability to Prepare/Respond/Recover | People with more dependents may struggle to respond to and recover from extreme climatic events. |
| Low Skilled Employment | Income | Adaptive Capacity – Ability to Prepare/Respond/Recover | People in low skills employment are likely to have less income, reducing their ability to adapt before, during, or after an event. |
| Unemployment | Income | Adaptive Capacity – Ability to Prepare/Respond/Recover | Unemployed persons are more likely to have less incomes, and have limited ability to make physical adjustments to their property to adapt to flooding |
| Population with no higher education | Information Access/Use | Adaptive Capacity – Ability to Prepare/Respond/Recover | People with no formal education may find it difficult to interpret and/or act up on information received |
| Population who do not speak English well or at all | Information Access/Use | Adaptive Capacity – Ability to Prepare/Respond/Recover | People with poor English ability may  find it difficult to interpret and/or act up on information received |
| Persons with an Intellectual or Learning Disability | Information Access/Use | Adaptive Capacity – Ability to Prepare/Respond/Recover | People with an iintellectual or learning disability may  find it difficult to interpret and/or act up on information received |
| New residents | Local Knowledge | Adaptive Capacity – Ability to Prepare/Respond/Recover | Residents living in the area less than a year are likely to have less local knowledge and may be less aware of hazards. |
| Foreign nationals – Born outside Northern Ireland /UK or Republic of Ireland | Local Knowledge | Adaptive Capacity – Ability to Prepare/Respond | Foreign nationals are likely to have less local knowledge and may be less aware of hazards. |
| Households with no motor car/van | Mobility | Adaptive Capacity – Ability to Respond/Recover | No personal access to a vehicle may restrict evacuation during a flooding event |
| % Dependent population | Mobility | Adaptive Capacity – Ability to Respond/Recover | A high dependent population may make short-term evacuation of people and possessions more difficult |
| Persons living in a non-urban area | Physical Access | Adaptive Capacity – Ability to Respond | People in rural areas are likely in areas with low service provision |
| Travel distance | Physical Access | Adaptive Capacity – Ability to Respond | People who have to travel more than 20km to work are likely to be living in areas with low service provision |
| Primary School Age Children | Social Network | Adaptive Capacity – Ability to Respond/Recover | Those with primary school age children tend to have a stronger social network that can assist them during a flooding event. |
| Households with one person | Social Network | Adaptive Capacity – Ability to Respond/Recover | Those living alone likely lack a support network that can assist them during a flooding event. |
| Households with no central heating | Housing Characteristics | Adaptive Capacity – Ability to Recover | People that reside in households with no central heating may struggle to recover from the impacts of flooding events (cold and damp) |
| Households renting | Tenure | Adaptive Capacity – Ability to Prepare | Renters are more likely to have lower incomes, plus have limited ability to make physical adjustments to their property to adapt to flooding. |
| Impervious Surface | Physical Environment | Enhanced Exposure | Increased area of impervious surface enhances the impacts of flooding |
| Tree Cover | Physical Environment | Enhanced Exposure | Increased area of tree cover and greenspace reduces the impacts of flooding |
| Households that are caravans/mobile/temporary structures | Housing Characteristics | Enhanced Exposure | Caravans/mobile homes may not offer as much protection to residents as compared to permanent housing |

*Table 12: Indicators used in Rimini, Italy to create an SVI*

| **Indicator** | **Domain** | **Dimension** | **Rationale** |
| --- | --- | --- | --- |
| Boys under 5 years of age | Age | Sensitivity | Physiologically, the young have a greater susceptibility to the effects of extreme heat. |
| Girls under 5 years of age | Age | Sensitivity | Physiologically, older people have a greater susceptibility to the effects of extreme heat. |
| Males over 75 years of age | Age | Sensitivity | Physiologically, older people have a greater susceptibility to the effects of extreme heat. |
| Females over 75 years of age | Age | Sensitivity | Physiologically, older people have a greater susceptibility to the effects of extreme heat. |
| Dependents Rate | Income | Adaptive Capacity – Ability to Prepare/Respond/Recover | People with dependents may struggle to respond to and recover from extreme climatic events |
| Unemployment | Income | Adaptive Capacity – Ability to Prepare/Respond/Recover | Unemployed persons are more likely to have less incomes, and have limited ability to make physical adjustments to their property to adapt to extreme heat |
| Population with no higher education | Information Access/Use | Adaptive Capacity – Ability to Prepare/Respond/Recover | People with no formal education may find it difficult to interpret and/or act up on information received |
| Percentage of foreign nationals | Local Knowledge | Adaptive Capacity – Ability to Prepare/Respond | Foreign nationals are likely to have less local knowledge and be less aware of hazards |
| Primary School Age Children | Social Network | Adaptive Capacity – Ability to Respond/Recover | Those with primary school age children tend to have a stronger social network that can assist them during a heatwave event |
| Households with one person | Social Network | Adaptive Capacity – Ability to Respond/Recover | Those living alone likely lack a support network that can assist them during a heatwave event |
| Impervious Surface | Physical Environment | Enhanced Exposure | Increased area of impervious surface enhances the urban heat island affect |
| Tree Cover | Physical Environment | Enhanced Exposure | Increased area of tree cover and greenspace reduces the urban heat island affect |

**References**

[1] J.M. Fitton, B. O’Dwyer, B. Maher, Developing a social vulnerability to environmental hazards index to inform climate action in Ireland. Irish Geography, 54(2) (2021) DOI: 10.2014/igj.v54i2.1468

[2] E. Mavhura, B. Manyena, A.E. Collins, An approach for measuring social vulnerability in context: The case of flood hazards in Muzarabani district, Zimbabwe. Geoforum, 86 (2017) 103-117.

[3] A. Kazmierczak, G. Cavan, A. Connelly, S. Lindley, Mapping flood disadvantage in Scotland 2015 (2015) Edinburgh: Scottish Government.

[4] S. Lindley, J. O’Neill, J. Kandeh, N. Lawson, R. Christian, M. O’Neill, Climate change, justice and vulnerability. Joseph Rowntree Foundation, York, (2011) 1-177.

[5] S. L. Cutter, C.T. Emrich, J.J. Webb, D. Morath, Social vulnerability to climate variability hazards: A review of the literature. Final Report to Oxfam America, 5 (2009) 1-44.

[6] J. Dong, J. Peng, X. He, J. Corcoran, S. Qiu, X. Wang, Heatwave-induced human health risk assessment in megacities based on heat stress-social vulnerability-human exposure framework. Landscape and Urban Planning, 203, (2020) 103907.

[7] G. D. Kumar, K.C. Pradhan, Assessing the district-level flood vulnerability in Bihar, eastern India: an integrated socioeconomic and environmental approach. Environmental Monitoring and Assessment, 196(9) (2024a) 799.

[8] G. Oulahen, D. Shrubsole, G. McBean, Determinants of residential vulnerability to flood hazards in Metro Vancouver, Canada. Natural Hazards, 78 (2015) 939-956.

[9] I. Isia, T. Hadibarata, R.I. Hapsari, M.N.H. Jusoh, R.K. Bhattacharjya, N.F. Shahedan, Assessing social vulnerability to flood hazards: a case study of Sarawak's divisions. International journal of disaster risk reduction, 97 (2023) 104052.

[10] S. Rufat, E. Tate, C.T. Emrich, F. Antolini, How valid are social vulnerability models?. Annals of the American Association of Geographers, 109(4) (2019) 1131-1153.

[11] S.L. Cutter, B.J. Boruff, W.L. Shirley, Social Vulnerability to Environmental Hazards. *Social Science Quarterly*, *84*(2) (2003) 242–261.

[12] S. L. Cutter, C. Finch, Temporal and spatial changes in social vulnerability to natural hazards, Proceedings of the National Academy of Sciences of the United States of America, 105(7) (2008) 2301–6. doi: 10.1073/pnas.0710375105.

[13] S. Greiving, M. Fleischhauer, J. Lückenkötter, A methodology for an integrated risk assessment of spatially relevant hazards. Journal of environmental planning and management, 49(1) (2006) 1-19.

[14] A. Hebb, L. Mortsch, Floods: Mapping vulnerability in the Upper Thames watershed under a changing climate. Project Report XI, University of Waterloo, (2007) 1-53.

[15] J. Andrey, B. Jones, The dynamic nature of social disadvantage: Implications for hazard exposure and vulnerability in Greater Vancouver. The Canadian Geographer/Le Géographe Canadien, 52 (2) (2008) 146-168.

[16] T.W. Collins, S.E. Grineski, M.D.L.R. Aguilar, Vulnerability to environmental hazards in the Ciudad Juárez (Mexico)–El Paso (USA) metropolis: a model for spatial risk assessment in transnational context. Applied Geography, 29(3) (2009) 448-461.

[17] D. Filiberto, E. Wethington, K. Pillemer, N. Wells, M. Wysocki, J.T. Parise, Older people and climate change: Vulnerability and health effects. Generations, 33(4) (2009) 19-25.

[18] S.G. Nayak, S. Shrestha, P.L. Kinney, Z. Ross, S.C. Sheridan, C.L. Pantea,.... S.A. Hwang, Development of a heat vulnerability index for New York State. Public health, 161 (2018) 127-137.

[19] E. Tate, Uncertainty analysis for a social vulnerability index. Annals of the association of American geographers, 103(3) (2013) 526-543.

[20] S. Bjarnadottir, Y. Li, M.G. Stewart, Social vulnerability index for coastal communities at risk to hurricane hazard and a changing climate. Natural Hazards, 59, (2011) 1055-1075.

[21] M. Wu, M. Chen, G. Chen, D. Zheng, Y. Zhao, X. Wei, Y. Xin, Research on methodology for assessing social vulnerability to urban flooding: A case study in China. Journal of Hydrology, 645 (2024) 132177.

[22] L. Chakraborty, H. Rus, D. Henstra, J. Thistlethwaite, D. Scott, A place-based socioeconomic status index: Measuring social vulnerability to flood hazards in the context of environmental justice. International Journal of Disaster Risk Reduction, 43, (2020) 101394.

[23] F. Cian, C. Giupponi, M. Marconcini, Integration of earth observation and census data for mapping a multi-temporal flood vulnerability index: a case study on Northeast Italy. Natural Hazards, 106, (2021) 2163-2184.

[24] C. Milton, Measuring vulnerability to flooding using two indices: A case study of Miami-Dade County, Florida (2021) (Master's thesis, University of Twente).

[25] S. Y. Wu, B. Yarnal, A. Fisher, Vulnerability of coastal communities to sea-level rise: a case study of Cape May County, New Jersey, USA. Climate research, 22(3) (2002) 255-270.

[26] L. Chakraborty, J. Thistlethwaite, D. Henstra, Flood vulnerability and climate change: Improving flood risk assessment by mapping socioeconomic vulnerability in a mid-sized Canadian city. Canadian Climate Institute (2021).

[27] M. Preisser, P. Passalacqua, R.P. Bixler, S. Boyles, A network-based analysis of critical resource accessibility during floods. Frontiers in Water, 5 (2023) 1278205.

[28] B.H. Narendra, C.A. Siregar, I.W.S. Dharmawan, A. Sukmana, A. Pratiwi, I.B. Pramono ... T.W. Yuwati, A review on sustainability of watershed management in Indonesia. Sustainability, 13(19) (2021) 11125.

[29] L. Chakraborty, J. Thistlethwaite, D. Scott, D. Henstra, A. Minano, H. Rus, Assessing social vulnerability and identifying spatial hotspots of flood risk to inform socially just flood management policy. Risk Analysis, 43(5) (2023) 1058-1078.

[30] J. Meadows, A. Mansour, M.R. Gatto, A. Li, A. Howard, R. Bentley, Mental illness and increased vulnerability to negative health effects from extreme heat events: a systematic review. Psychiatry research, 332 (2024) 115678.

[31] S. Rufat, E. Tate, C.G. Burton, A.S. Maroof, Social vulnerability to floods: Review of case studies and implications for measurement. International journal of disaster risk reduction, 14, (2015) 470-486.

[32] T. W. Collins, S.E. Grineski, J. Chakraborty, A.B.Flores, Environmental injustice and Hurricane Harvey: A household-level study of socially disparate flood exposures in Greater Houston, Texas, USA. Environmental research, 179 (2019) 108772.

[33] M. B. Hahn, A. M. Riederer, S.O. Foster, The Livelihood Vulnerability Index: A pragmatic approach to assessing risks from climate variability and change—A case study in Mozambique. Global environmental change, 19(1) (2009) 74-88.

[34] L.T. Mazumder, S. Landry, K. Alsharif, Coastal cities in the Southern US floodplains: An evaluation of environmental equity of flood hazards and social vulnerabilities. Applied geography, 138 (2022) 102627.

[35] P. Sayers, S. Lindley, S. Carr, R. Figueroa-Alfaro, The impacts of climate change on population groups in Scotland. (2023) Sayers and Partners.

[36] D.J. Odeh, Natural hazards vulnerability assessment for statewide mitigation planning in Rhode Island. Natural Hazards Review, 3(4) (2002) 177-187.

[37] B. Jones, J. Andrey, Vulnerability index construction: methodological choices and their influence on identifying vulnerable neighbourhoods. International journal of emergency management, 4(2) (2007) 269-295.

[38] M. B. LaLone, Neighbors Helping Neighbors: An Examination of the Social Capital Mobilization Process for Community Resilience to Environmental Disasters. Journal of Applied Social Science, 6(2) (2012) 209-237.

[39] J. Flaherty, R.B. Brown, A multilevel systemic model of community attachment: Assessing the relative importance of the community and individual levels. American Journal of Sociology, 116(2) (2010) 503-542.

[40] S.A. Martin, A framework to understand the relationship between social factors that reduce resilience in cities: Application to the City of Boston. International journal of disaster risk reduction, 12 (2015) 53-80.

[41] M. Domínguez, D. Montolio, Bolstering community ties as a mean of reducing crime. Journal of Economic Behavior & Organization, 191 (2021) 916-945.

[42] N. Gallent, The social value of second homes in rural communities. Housing, Theory and Society, 31(2) (2014) 174-191.

[43] G. D. Roude, K. Wu, L. Richardson, A. Tucker, L. Moss, M. Kondo, .... K.P. Theall, The Impact of Vacant and Abandoned Property on Health and Well-Being: A Qualitative Inquiry. Applied Research in Quality of Life, (2024) 1-21.

[44] F. Li, T. Yigitcanlar, M. Nepal, K. Nguyen, F. Dur, W. Li, Assessing heat vulnerability and multidimensional inequity: Lessons from indexing the performance of Australian capital cities. Sustainable Cities and Society, 115, (2024) 105875.

[45] S. Sabrin, M. Karimi, R. Nazari, Modeling heat island exposure and vulnerability utilizing earth observations and social drivers: A case study for Alabama, USA. Building and Environment, 226 (2022) 109686.

[46] A. Kumar, S. Mondal, P. Lal, Analysing frequent extreme flood incidences in Brahmaputra basin, South Asia. Plos one, 17(8) (2022) e0273384.

[47] P. Kumar, S.E. Debele, S. Khalili, C.H. Halios, J. Sahani, N. Aghamohammadi, ... L. Jones, Urban heat mitigation by green and blue infrastructure: Drivers, effectiveness, and future needs. The Innovation, 5(2) (2024b).

[48] D. P. Johnson, A. Stanforth, V. Lulla, G. Luber, Developing an applied extreme heat vulnerability index utilizing socioeconomic and environmental data. Applied Geography, 35(1-2) (2012) 23-31.

[49] S. Mishra, D. Suar, Do lessons people learn determine disaster cognition and preparedness?. Psychology and Developing Societies, 19(2) (2007) 143-159.

[50] R. Chicas, N. Xiuhtecutli, N.E. Dickman, M.L. Scammell, K. Steenland, V.S. Hertzberg, L. McCauley, Cooling intervention studies among outdoor occupational groups: A review of the literature. American journal of industrial medicine, 63(11) (2020) 988-1007.

[51] T. L. Chen, H. Lin, Y.H. Chiu, Heat vulnerability and extreme heat risk at the metropolitan scale: A case study of Taipei metropolitan area, Taiwan. Urban Climate, 41, (2022) 101054.

[52] M. Zsamboky, A. Fernández-Bilbao, D. Smith, J. Knight, J. Allan, Impacts of climate change on disadvantaged UK coastal communities. Joseph Rowntree Foundation, (2011) 1-63.

[53] J. Bao, X. Li, C. Yu, The construction and validation of the heat vulnerability index, a review. International journal of environmental research and public health, 12 (7) (2015) 7220-7234.

[54] I. García, N. Hernandez, N. “They're just trying to survive”: The relationship between social vulnerability, informal housing, and environmental risks in Loíza, Puerto Rico, USA. World Development Sustainability, 2 (2023) 100062.

[55] A.H. Thieken, M. Muller, H. Kreibich, B. Merz, Flood damage and influencing factors: New insights from the August 2002 flood in Germany. Water Resources Research 41 (2005). doi:10.1029/2005WR004177.

[56] I. Ajtai, H. Ștefănie, C. Maloș, C. Botezan, A. Radovici, M. Bizău-Cârstea, C. Baciu, Mapping social vulnerability to floods. A comprehensive framework using a vulnerability index approach and PCA analysis. Ecological Indicators 154 (2023) 110838.

[57] A. Scolobig, B. De Marchi, M. Borga, The missing link between flood risk awareness and preparedness: findings from case studies in an Alpine Region. Natural hazards, 63 (2012) 499-520.

[58] E. Ridolfi, F. Albrecht, G. Di Baldassarre, Exploring the role of risk perception in influencing flood losses over time. Hydrological Sciences Journal, 65(1) (2020) 12-20.

[59] K. Bradford, L. Abrahams, M. Hegglin, K. Klima, A heat vulnerability index and adaptation solutions for Pittsburgh, Pennsylvania. Environmental science & technology, 49(19) (2015) 11303-11311.

[60] J. Qi, L. Ding, S. Lim, Toward cool cities and communities: A sensitivity analysis method to identify the key planning and design variables for urban heat mitigation techniques. Sustainable Cities and Society, 75 (2021) 103377.

[61] K. C. Conlon, E. Mallen, C.J. Gronlund, V.J. Berrocal, L. Larsen, M.S. O’Neill, Mapping human vulnerability to extreme heat: A critical assessment of heat vulnerability indices created using principal components analysis. Environmental health perspectives, 128(9) (2020) 097001.

[62] Y. Xu, T. Hong, W. Zhang, Z. Zeng, M. Wei, Heat vulnerability index development and mapping (2021).

[63] L. Chapman, J.A. Azevedo, T. Prieto-Lopez, Urban heat & critical infrastructure networks: A viewpoint. Urban Climate, 3 (2013) 7-12.

[64] G. Forzieri, A. Bianchi, F.B. Silva, M.A.M. Herrera, A. Leblois, C. Lavalle, ... L. Feyen, Escalating impacts of climate extremes on critical infrastructures in Europe. Global environmental change, 48 (2018) 97-107.

[65] L. McColl, E.J. Palin, H.E. Thornton, D.M. Sexton, R. Betts, K. Mylne, Assessing the potential impact of climate change on the UK’s electricity network. Climatic change, 115 (2012) 821-835.

[66] D. McEvoy, I. Ahmed, J. Mullett, The impact of the 2009 heat wave on Melbourne's critical infrastructure. Local environment, 17(8) (2012) 783-796.

[67] A. Mavrogianni, M. Davies, J. Taylor, Z. Chalabi, P. Biddulph, E. Oikonomou, ... B. Jones, The impact of occupancy patterns, occupant-controlled ventilation and shading on indoor overheating risk in domestic environments. Building and Environment, 78 (2014) 183-198.

[68] H. Tan, R. Kotamarthi, J. Wang, Y. Qian, T.C. Chakraborty, Impact of different roofing mitigation strategies on near-surface temperature and energy consumption over the Chicago metropolitan area during a heatwave event. Science of the Total Environment, 860 (2023) 160508.

[69] L. Inostroza, M. Palme, F. De La Barrera, A heat vulnerability index: spatial patterns of exposure, sensitivity and adaptive capacity for Santiago de Chile. *PLOS one*, *11*(9) (2016) e0162464.
